# Proteomic analysis of protein composition of rat hippocampus exposed to morphine for 10 days; comparison with animals after 20 days of morphine withdrawal

**DOI:** 10.1371/journal.pone.0231721

**Published:** 2020-04-15

**Authors:** Hana Ujcikova, Kristina Cechova, Michal Jagr, Lenka Roubalova, Miroslava Vosahlikova, Petr Svoboda

**Affiliations:** 1 Laboratory of Membrane Receptors, Department of Biomathematics, Institute of Physiology of the Czech Academy of Sciences, Prague 4, Czech Republic; 2 Department of Biochemistry, Faculty of Science, Charles University in Prague, Prague 2, Czech Republic; 3 Laboratory of Analysis of Biologically Important Compounds, Institute of Physiology of the Czech Academy of Sciences, Prague 4, Czech Republic; University of Arizona College of Medicine, UNITED STATES

## Abstract

Opioid addiction is recognized as a chronic relapsing brain disease resulting from repeated exposure to opioid drugs. Cellular and molecular mechanisms underlying the ability of organism to return back to the physiological norm after cessation of drug supply are not fully understood. The aim of this work was to extend our previous studies of morphine-induced alteration of rat forebrain cortex protein composition to the hippocampus. Rats were exposed to morphine for 10 days and sacrificed 24 h (groups +M10 and −M10) or 20 days after the last dose of morphine (groups +M10/−M20 and −M10/−M20). The six altered proteins (≥2-fold) were identified in group (+M10) when compared with group (−M10) by two-dimensional fluorescence difference gel electrophoresis (2D-DIGE). The number of differentially expressed proteins was increased to thirteen after 20 days of the drug withdrawal. Noticeably, the altered level of α-synuclein, β-synuclein, α-enolase and glyceraldehyde-3-phosphate dehydrogenase (GAPDH) was also determined in both (±M10) and (±M10/−M20) samples of hippocampus. Immunoblot analysis of 2D gels by specific antibodies oriented against α/β-synucleins and GAPDH confirmed the data obtained by 2D-DIGE analysis. Label-free quantification identified nineteen differentially expressed proteins in group (+M10) when compared with group (−M10). After 20 days of morphine withdrawal (±M10/−M20), the number of altered proteins was increased to twenty. We conclude that the morphine-induced alteration of protein composition in rat hippocampus after cessation of drug supply proceeds in a *different manner* when compared with the forebrain cortex. In forebrain cortex, the total number of altered proteins was decreased after 20 days without morphine, whilst in hippocampus, it was increased.

## Introduction

Opioid dependence and withdrawal syndrome are the leading problems associated with licit and illicit opioid use. In spite of the large literature on opioid addiction, there is only little available information about the durability and reversibility of potentially adverse effects of opioid treatment and withdrawal. From a clinical point of view, drug withdrawal is the leading pathophysiological state driving opioid dependence and addictive behaviors [1,2]. Here, we focused our attention on morphine as the *prototypical opioid agonist with which all others are compared*.

Our previous results, in accordance with the data of Sim et al. [3], Sim-Selley et al. [4] and Maher et al. [5], indicated a desensitization of (2-D-alanine2-4-methylphenylalanine-5-glycineol)-enkephalin (DAMGO)- and (2-D-alanine-5-D-leucine)-enkephalin (DADLE)-stimulated G-protein responses in plasma membranes (PM) isolated from forebrain cortex (FBC) of rats exposed to morphine for 10 days [6]. Behavioral tests proved that these rats developed a *tolerance* to this drug as there was no significant difference between control (−M10) and morphine-treated (+M10) rats in the sensitivity to heat stimulation (*hot-plate test*), which was determined as a delay in hind paw withdrawal. Tolerance to morphine in (+M10) rats was also evidenced by *hind paw withdrawal* test. Precipitation of morphine *withdrawal state* by naloxone resulted in a rapid and dramatic opiate abstinence syndrome. There were no detectable signs of abstinence syndrome (such as body shakes, teeth clatter, vacuous chewing, ptosis, irritability to touch, diarrhea), in the (−M10) animals.

In opioid-dependent subjects, the body has adapted to perpetually high opioid tone by making *homeostatic* adjustments in anti-opioid systems [7]. In an effort to clarify biochemical mechanisms of development of opiate tolerance and dependence, our previous results showed that in rat FBC, such homeostatic adjustments were accompanied by a reversible and specific up-regulation of adenylyl cyclases I and II (ACI and ACII). Importantly, the up-regulation of ACI and ACII disappeared after 20 days of morphine withdrawal (±M10/−M20) [8,9].

Proteomic analyses of the consequences of a 10-day morphine treatment and subsequent 20-day drug withdrawal on FBC, which were based on CBB-staining of 2D gels and matrix-assisted laser desorption/ionization time-of-flight mass spectrometry (MALDI-TOF MS/MS), showed that the number of altered proteins was *decreased* from 28 (determined in ±M10 rats) to 14 (determined in ±M10/−M20 rats). When using label-free quantification (LFQ), the number of altered proteins was *decreased* from 113 to 19 [10]. Thus, we brought a straightforward evidence for the ability of the organism to oppose the drastic, morphine-induced change of the target tissue protein composition with the aim to return to the physiological norm after a complete removal of the drug.

The chronic administration of opioid drugs was reported to modulate synaptic transmission and plasticity of hippocampus and inhibit adult neurogenesis [11]. Chronic opioid administration also resulted in obtuseness of spatial memory and increase in the expression of proteins functionally associated with apoptosis and neurotoxicity [12]. In contrast to chronic opioid treatment, opioid withdrawal was associated with enhanced hippocampal plasticity [13]. Interestingly in the context with studies of prolonged morphine effect on the brain, the chronic antidepressant treatment was found to increase neurogenesis in adult rat hippocampus [14].

As *neurodegeneration* was frequently described as a pathological state accompanying addiction to opioid drugs and hippocampus is regarded as one of the key brain areas [15,16], together with striatum [17] and cerebellum [18], which exhibit *neurogenesis* in adult brain, the aim of this work was to extend our previous proteomic studies of morphine-induced alteration of rat forebrain cortex protein composition to the hippocampus.

The two-dimensional fluorescence difference gel electrophoresis (2D-DIGE) and MALDI-TOF MS/MS was used for comparison of protein profiling in hippocampal samples prepared from (+M10), (−M10), (+M10/−M20) and (−M10/−M20) groups of rats, [19,20]. Results obtained by 2D-DIGE were verified by immunoblot analysis of 2D gels and a gel-free, label free proteomic analysis, MaxLFQ [21].

## Materials and methods

### Chemicals

Acrylamide and bis-acrylamide were from SERVA (Heidelberg, Germany). Immobiline DryStrips, Pharmalyte buffer (broad pH range 3–10), Immobiline DryStrip cover fluid and Amersham^™^ CyDye DIGE Fluors (minimal dyes) for 2D-DIGE were purchased from GE Healthcare (Piscataway Township, NJ). Complete protease inhibitor cocktail was from Roche Diagnostic (Mannheim, Germany). All others chemicals were of the highest purity available and purchased from Sigma-Aldrich (St. Louis, USA).

### Antibodies

The α- and β-synucleins were identified by mouse monoclonal antibody purchased from Santa Cruz, Inc. (Dallas, USA): α/β-synuclein (F-11, sc-514908, 1:500 dilution). glyceraldehyde-3P-dehydrogenase (GAPDH)- and actin-oriented antibodies were also from Santa Cruz, Inc. (Dallas, USA): GAPDH (FL-335, sc-25778, 1:5000 dilution), actin (I-19, sc-1616, 1:2500 dilution). Beta-actin polyclonal antibody was purchased from Bioss Antibodies: β-actin (bs-0061R, 1:5000 dilution). Sheep anti-mouse IgG-HRP (NA931V, 1:10000 dilution) was from GE Healthcare UK and goat anti-rabbit IgG-HRP (sc-2004, 1:10000 dilution) was from Santa Cruz, Inc. (Dallas, USA).

### Morphine treatment of experimental animals

Young adult morphine-naive male Wistar rats (220–250 g) were exposed to increasing doses of morphine (10–40 mg/kg) dissolved in 0.9% NaCl for 10 consecutive days as described before [8,10,22]. Food (Altromin standard diet, Germany) and drinking water was provided *ad libitum*. The morphine-treated rats were sacrificed 24 h (group +M10) or 20 days (group +M10/−M20) after the last dose of morphine. Control animals (−M10, −M10/−M20) received 0.9% NaCl for 10 days and were sacrificed in parallel with morphine-treated rats.

Rats were killed by decapitation under ether narcosis, hippocampi dissected, separated from other parts of brain, washed from remaining blood, snap frozen in liquid nitrogen and stored at −80 °C until use. The experiments were approved by Animal Care and Use Committee of the Institute of Physiology of the Czech Academy of Sciences to be in agreement with Animal Protection Law of the Czech Republic as well as the European Communities Council Directive (86/609/EEC).

### Preparation of post-nuclear supernatant fraction (PNS) from rat hippocampus

The hippocampal tissue pieces were minced, diluted in 250 mM sucrose, 20 mM Tris-HCl, 3 mM MgCl_2_, 1 mM EDTA (pH 7.6) containing protease inhibitor cocktail and fresh phenylmethylsulfonyl fluoride (1 mM final concentration), homogenized for 5 min (2 g w. w. per 10 ml) and centrifuged for 5 min (1200 × *g*). The 1200 × *g* supernatant represented the PNS fraction.

### 2D-DIGE

Samples of PNS containing 1 mg protein were precipitated with ice-cold acetone overnight at– 20 °C. After centrifugation at 9000 × *g* for 20 min at 4 °C, the supernatant was removed and the pellet was extracted with ice-cold 6% trichloroacetic acid (TCA) for 1.5 h on ice. After centrifugation at 9000 × *g* for 10 min at 4 °C, the supernatant was discarded and the pellet washed with 600 μl of ice-cold 96% ethanol for 1 h at room temperature. The mixture was centrifuged at 9000 × *g* for 10 min at 4 °C and the remaining pellet was solubilized with 600 μl lysis buffer containing 30 mM Tris, 7 M urea, 2 M thiourea, 4% (w/v) 3-[(3-cholamidopropyl) dimethylammonio]-1-propanesulfonate (CHAPS), pH was adjusted to 8.5 with 50 mM NaOH. Each 50 μg of protein sample (−M10, +M10 and −M10/−M20, +M10/−M20) was labeled with 600 pmol of cyanine CyDye DIGE Fluor minimal dyes Cy3, Cy5 or Cy2 according to Jágr et al. [20].

In the first set of experiments, samples from (+M10) group were labeled with Cy3 and samples from control group (−M10) were labeled with Cy5. In the second set of experiments, samples prepared from rats sacrificed 20 days after morphine withdrawal (+M10/−M20) were labeled with Cy3; samples from the control group (−M10/−M20) were labeled with Cy5. Cy2 was always used to label a mixture of equal amounts of protein taken from all samples of PNS. Cy2-labeled protein mix thus represented an internal standard.

Samples were briefly vortexed and incubated on ice for 30 min in the dark. Staining was terminated by the addition of 10 mM lysine for 10 min on ice in the dark. Dye-labeled PNS samples were combined in such a way that each mixture was comprised of protein samples from (+M10, −M10) or (+M10/−M20, −M10/−M20) groups plus the aliquot of the internal standard (1:1:1, v/v/v). Finally, the mixture of labeled samples was mixed with an equal amount of sample buffer (8 M urea, 130 mM dithiothreitol (DTT), 4% (w/v) CHAPS, 2% (v/v) BioLyte 3–10 buffer (Bio-Rad).

All the samples (250 μl) were then transferred into a groove of the Immobiline DryStrip Reswelling Tray (GE Healthcare). Immobiline DryStrips (linear pH gradient 3–11 NL, 13 cm) were placed into the protein samples and rehydrated overnight. The exposure of protein labeled with CyDyes to all light sources was kept to a minimum. Isoelectric focusing was performed using the Multiphor II system (GE Healthcare) at 14 °C in the following manner: 150 V for 5 h, 500 V for 1 h, 3500 V for 12 h and 500 V for 3 h. The focused strips were stored at– 20 °C or immediately used.

The strips were rinsed thoroughly with ultrapure water, dried quickly on filter paper and equilibrated in 5 ml of equilibration buffer (50 mM Tris-HCl pH 6.8, 6 M urea, 0.1 mM EDTA, 2% SDS, 30% glycerol and 0.01% bromophenol blue) containing 1% DTT for 10 min in order to reduce disulfide bridges and other oxidized groups. Subsequently, the strips were alkylated in equilibration buffer containing 2.5% iodoacetamide for 10 min. Molecular weight markers were loaded onto a piece of filter paper and placed close to the alkaline side of the strip. The strip and molecular marker were covered with 0.5% agarose. Gels were run vertically at a constant current of 20 mA for 20 min and then at 90 mA for 4 h till the bromophenol blue dye reached the end of the gel. The apparatus was cooled to 15 °C using the Hoefer SE 600 unit (GE Healthcare). All the 2D-DIGE analyses were performed three times. After electrophoresis, the gels were washed with ultrapure water for 2×15 min before scanning.

### 2D image analysis

Three different gel images were obtained from one gel at the appropriate wavelength. They are Cy2 (blue 488 nm laser and 520 nm band pass emission filter), Cy3 (green 532 nm laser and 580 nm band pass emission filter) and Cy5 (red 633 nm laser and 670 nm band pass emission filter) by using a Pharos FX^™^ scanner (Bio-Rad) at a resolution of 50 μm.

The analysis of DIGE gels was done using PDQuest^™^ software (Bio-Rad), version 7.3.1. and 8.0.1. Student´s *t*-test was used to calculate significant differences in relative abundances of protein spot features in the (−M10) vs. (+M10) or (−M10/−M20) vs. (+M10/−M20) PNS samples. Protein levels showing significant quantitative differences at least 2-fold (p≤0.05) were selected for mass spectrometric analysis. P-values were calculated via GraphPad*Prism4* software.

### Colloidal Coomassie staining

For MS analysis, the gels were stained by colloidal Coomassie brilliant blue G-250 (CBB) to enable the visual detection according to Fountoulakis et al. [23]. The fresh 2D gels were immediately fixed in 50% methanol/7% acetic acid for 1 h and incubated with CBB (17% ammonium sulfate, 34% methanol, 3% orthophosphoric acid and 0.1% CBB) overnight with gentle agitation. After staining, the gels were washed several times in ultrapure sterile water and stored in 1% acetic acid at 4 °C.

### MALDI-TOF MS/MS

Selected spots with significantly changed expression (≥2-fold) were cut out from 2D gels and processed as described previously [10,22]. Briefly, chopped 1x1x1 mm pieces were covered with 100 μl of 50 mM ammonium bicarbonate (ABC) buffer in 50% acetonitrile (ACN) (buffer A) with 50 mM DTT. After sonication, the supernatant was removed and each gel spot was mixed with 100 μl of buffer A with 50 mM iodoacetamide (IAA). After sonication, the supernatant was discarded and replaced with 100 μl of buffer A with 50 mM DTT. After sonication, the supernatant was discarded, and the samples were sonicated for 5 min in 100 μl of HPLC/MS-grade water. The water was then discarded, and the samples were again sonicated for 5 min in 100 μl of ACN. The ACN was removed, 5 ng of trypsin in 10 μl of 50 mM ABC was added to each sample, and the samples were incubated at 37 °C overnight. Trifluoroacetic acid (TFA) and ACN were added to reach final concentration of 1% TFA and 30% ACN. After sonication, 0.5 μl aliquot of trypsin digest was transferred onto MALDI target and let to dry. Subsequently, 0.5 μl drop of alpha-cyano-4-hydroxycinnamic acid solution (10 mg/ml in 50% ACN) was transferred onto MALDI target and let to dry again. Samples were measured using a 4800 Plus MALDI TOF/TOF analyzer (Applied Biosystems/MDS Sciex) equipped with a Nd:YAG laser (355 nm, firing rate 200 Hz).

The data were analyzed using in house running Mascot server 2.2.07 and matched against comprehensive UniProt database of protein sequences (27929 sequences; 14725510 residues). Database search criteria were as follows: enzyme = trypsin; taxonomy = *Rattus norvegicus*. Cystein carbamidomethylation was set as fixed modification, methionine oxidation and deamidation as variable modifications, respectively. Peptide mass tolerance was set to ±100 ppm, and fragment mass tolerance to ±0.4 Da with a maximum of two missed cleavages. Only hits that were scored as significant (MASCOT score ≥57, p<0.05) were accepted. All MALDI-TOF MS/MS analyses were performed in duplicates.

### 2D immunoblots

Samples of PNS containing 200–2000 μg of protein were prepared for isoelectric focusing as described above (2D-DIGE). Solubilization was performed with 250 μl IEF sample buffer containing 7 M urea, 2 M thiourea, 4% CHAPS, 1% DTT, 1% ampholines pH 3–10 and 0.01% bromophenol blue for 3 h at room temperature. Equilibration steps and SDS-PAGE was performed as described above (2D-DIGE).

After SDS-PAGE, the proteins were transferred to nitrocellulose (GE Healthcare, 1300 mA, 2.5 h) by using TE62 Standard Transfer Tank (Hoefer). All other steps were performed as described before [24]. The significance of the difference between (−M10) vs. (+M10) or (−M10/−M20) vs. (+M10/−M20) samples of PNS was analyzed by Student´s *t*-test and GraphPad*Prism4*. Results represent the average ± SEM.

### Label-free quantification (MaxLFQ)

The 200 μg of hippocampal PNS samples (+M10), (−M10), (+M10/−M20) and (−M10/−M20) were processed as described previously [10]. The 2 μg of the peptide mixture were loaded on trap column PepMap 300 C 18 column, 5 mm x 300 μm ID, 5 μm particles, 300 Å pore size (163589, Thermo Scientific). Individual peptides were separated by high-performance liquid chromatography (HPLC) using EASY-Spray column, 50 cm x 75 μm ID, PepMap C18, 2 μm particles, 100 Å pore size (ES 803, Thermo Scientific). The separation of peptides was achieved via a linear gradient for 2 hours between mobile phase A (2% acetonitrile, 0.1% formic acid) and B (80% acetonitrile, 0.1% formic acid). Separation was started by running the system with 2% mobile phase B, followed by gradient elution to 40% B.

All data were analyzed and quantified with MaxQuant software. The false discovery rate (FDR) was set to 1% for both proteins and peptides and a minimum length of seven amino acids was specified. The Andromeda search engine was used for the MS/MS spectra search against the UniProt *Rattus norvegicus* database of protein sequences. Enzyme specificity was set as C-terminal to Arg and Lys, also allowing cleavage at proline bonds and a maximum of two missed cleavages. Dithiomethylation of cysteine was selected as fixed modification and N-terminal protein acetylation and methionine oxidation as variable modifications. The “match between runs” feature of MaxQuant was used to transfer identifications to other LC-MS/MS runs based on their masses and retention time (maximum deviation 0.7 min) and this was also used in quantification experiments. Quantifications were performed with the label-free algorithms according to Cox et al. [21]. Binary logarithms of intensity ratios were then median calculated for each group and the difference between control and sample was determined. Only at least 1.7-fold significant differences calculated for at least 2 measured values from 3 replicates were taken into consideration.

### Protein determination

Lowry method was used for determination of protein concentration in all hippocampal samples of PNS (+M10, −M10, +M10/−M20 and −M10/−M20) using bovine serum albumin (Sigma, Fraction V) as a standard. Data were calculated by fitting the calibration curve as a quadratic equation.

## Results

### Comparative proteomic analysis of rat hippocampus from animals exposed to morphine for 10 days (+M10) and from control animals (−M10) by 2D-DIGE and MALDI-TOF MS/MS

**Fig 1:**
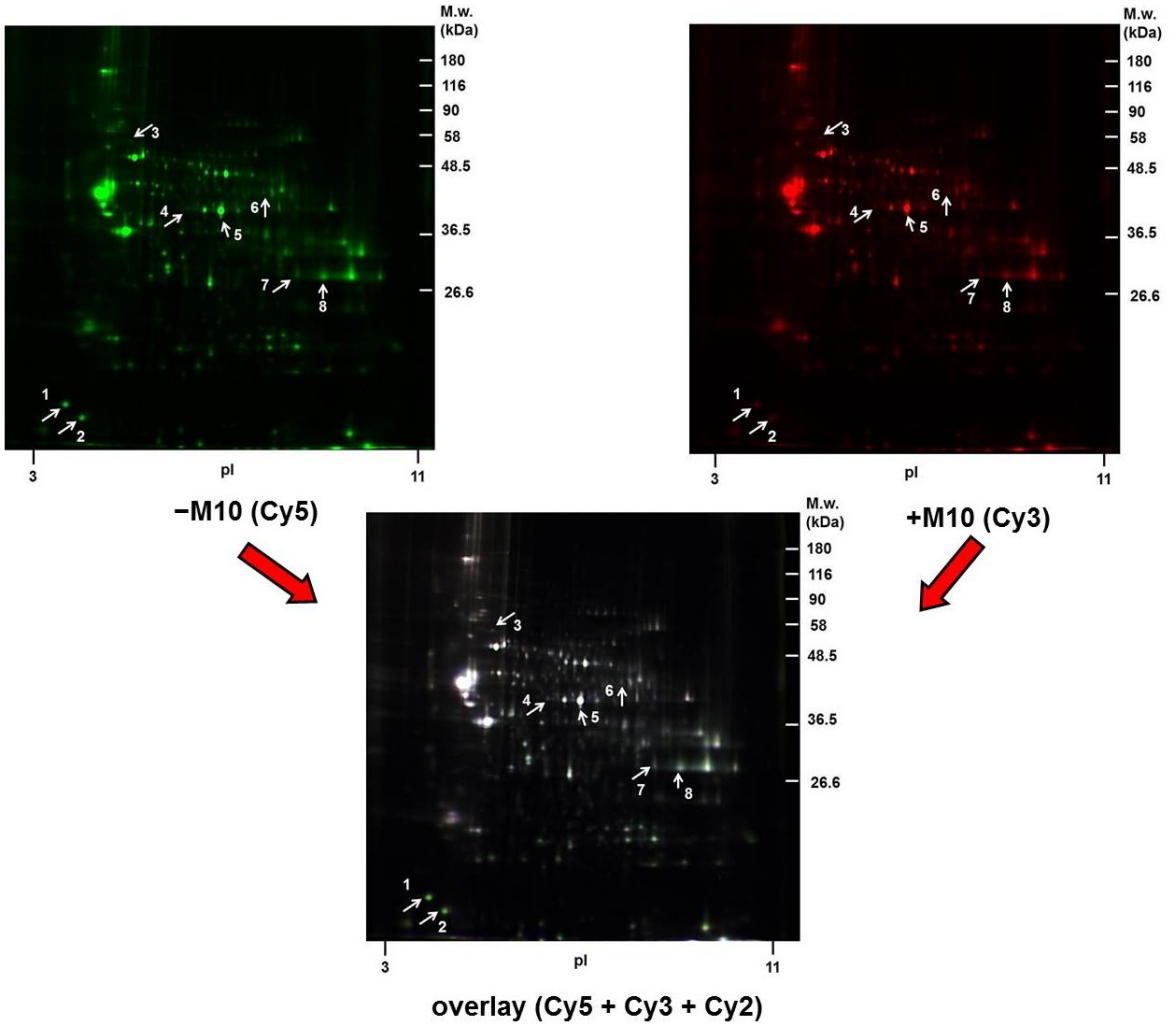
The 2D-DIGE comparative proteomic analysis of PNS fractions prepared from rat hippocampus of control (−M10) and morphine-treated rats (+M10) exposed to increasing doses of morphine for 10 days. PNS fractions were prepared as described in Methods. Staining of PNS samples and resolution of Cy5 (−M10)-, Cy3 (+M10)- and Cy2 (mixed)-labeled proteins in 2D gels (first dimension on pH 3–11 IPG strips and then by SDS-PADE on 10% acrylamide gels) was performed as described in Methods. White arrows indicate the protein spots altered at least 2-fold by morphine (p≤0.05). Comparison of DIGE gels was done by PDQuest^™^ (Bio-Rad). The significance of the difference between the three sets of (−M10) and (+M10) gels was determined by Student´s *t*-test. The positions of molecular weight markers are indicated on the right side and pI at the bottom of each gel.

**Fig 2:**
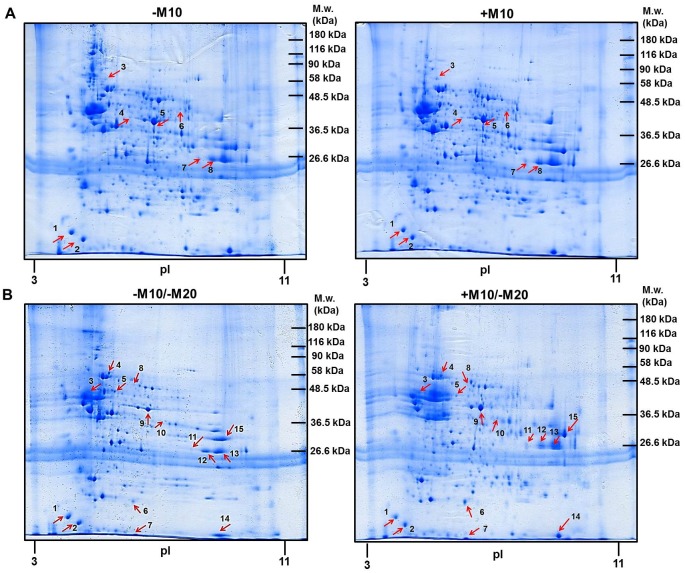
2D-ELFO of PNS hippocampal samples prepared from experimental groups (−M10) [a, upper left], (+M10) [a, upper right], (−M10/−M20) [b, lower left] and (+M10/−M20) [b, lower right] of control and morphine-treated rats. PNS fractions (2000 μg of protein) were resolved by 2D-ELFO and stained by CBB as described in Methods. After CBB staining, protein spots 1–8 (**A**, red arrows) and 1–15 (**B**, red arrows) were excised from gels and identified by MALDI-TOF MS/MS.

The (+M10) and (−M10) samples of PNS were labeled with Cy3 (+M10) and Cy5 (−M10), respectively, and resolved by 2D electrophoresis (2D-ELFO) together with internal standard (Cy2) as described in Methods. The total number of protein spots labeled with Cy3 (+M10) and Cy5 (−M10) was 307 (Fig 1). Among these, the 8 protein spots were recognized as significantly altered at least 2-fold (p≤0.05) when the difference between Cy3- and Cy5-stained gels was analyzed by PDQuest^™^ (Bio-Rad) and Student´s *t*-test (white arrows). These protein spots (1–8) were cut out from the CBB-stained gels (Fig 2A, upper panels) and identified by MALDI-TOF MS/MS (Table 1). Because the same proteins were detected in spots **4**, **5** (alpha-enolase) and **7**, **8** (glyceraldehyde-3-phosphate dehydrogenase), the number of significantly altered proteins (≥2-fold) in hippocampus by prolonged exposure of rats to morphine was 6 (complete list of peptides used for identification of altered proteins is presented in S1 Table).

**Table 1:** MALDI-TOF MS/MS analysis of eight altered protein spots in PNS prepared from hippocampus of rats exposed to morphine for 10 days and sacrificed 24 h after the last dose; *difference of protein composition in PNS samples prepared from groups (+M10) and (*−*M10)*.

Spot	Accession number	Protein name	Mascot score	Matched peptides	SC^a^ [%]	MW^b^ (kDa)	pI^c^	Change (fold)	p value
**1**	SYUB_RAT	Beta-synuclein	134	9	42	14.5	4.48	↓ 2.0	0.0060
**2**	SYUA_RAT	Alpha-synuclein	249	5	36	14.5	4.74	↓ 2.5	0.0251
**3**	ACTB_RAT	Actin, cytoplasmic 1 (fragment)	146	8	13	42.1	5.29	↑ 2.5	0.0068
**4**	ENOA_RAT	Alpha-enolase	117	6	11	47.4	6.16	↑ 2.2	0.0212
**5**	ENOA_RAT	Alpha-enolase	679	25	44	47.4	6.16	↑ 2.2	0.0073
**6**	DLDH_RAT	Dihydrolipoyl dehydrogenase, mitochondrial	180	7	12	54.6	7.96	↑ 2.0	0.0241
**7**	D3ZGY4_RAT	Glyceraldehyde-3-phosphate dehydrogenase	230	7	15	36.1	7.63	↓ 2.3	0.0106
**8**	D3ZGY4_RAT	Glyceraldehyde-3-phosphate dehydrogenase	306	13	18	36.1	7.63	↓ 2.3	0.0309

^*a*^ sequence coverage,

^*b*^ theoretical molecular weight,

^*c*^ theoretical isoelectric point; complete list of peptides used for identification is presented in S1 Table.

The presence of the same protein in more than just one spot, when resolved by 2D-ELFO, represents according to our previous experience [10,22], evidence for the existence of different subunits of the same protein. In general, this multiplicity occurs due to post-translational modifications of such proteins which result in the production of multiple spots when resolved in 2D gels [25].

**Fig 3:**
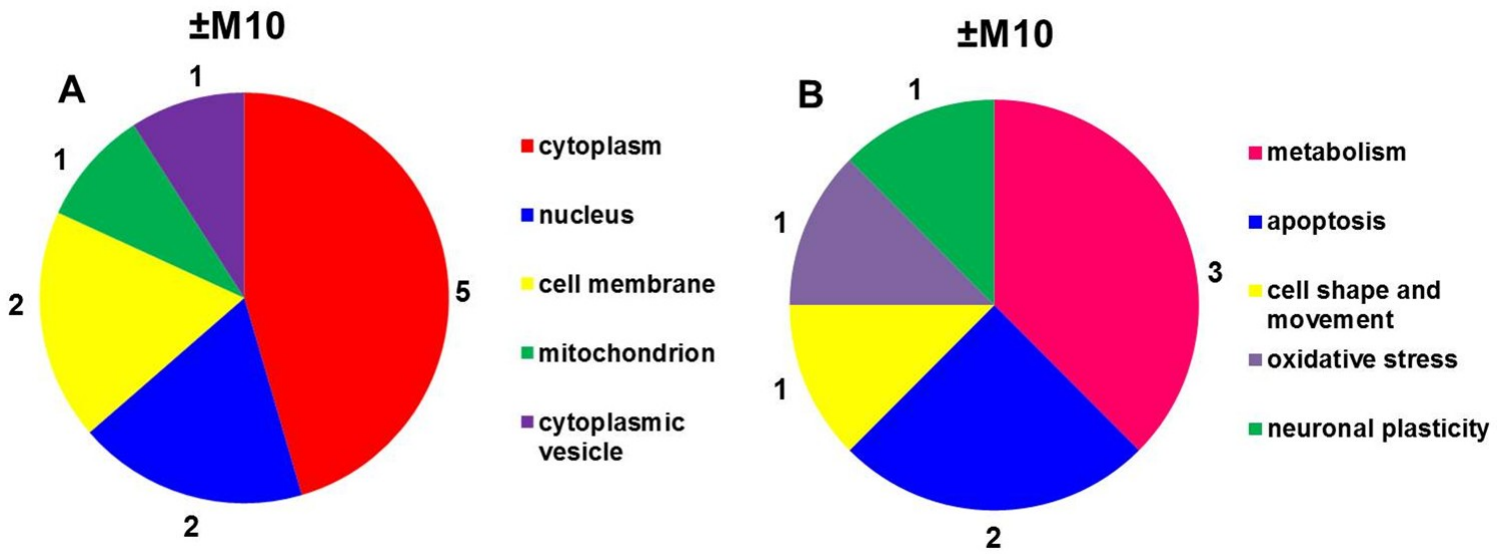
Subcellular localization (A) and functional significance (B) of morphine-altered proteins in experimental groups (+M10) and (−M10) identified by MALDI-TOF MS/MS.

The subcellular localization and functional significance of morphine-altered proteins was determined according to the current annotations in the UniProt database (Table 2, Fig 3). The altered proteins were found to be functionally related to *glycolysis*, *oxidative stress* and *apoptosis*: glyceraldehyde-3-phosphate dehydrogenase (spots **7**, ↓2.3-fold and **8**, ↓2.3-fold); *glycolysis*: alpha-enolase (spots **4**, ↑2.2-fold and **5**, ↑2.2-fold); *Krebs cycle*: mitochondrial dihydrolipoyl dehydrogenase (spot **6**, ↑2.0-fold,); *apoptosis*: α-synuclein (spot **2**, ↓2.5-fold); *cell motility*: cytoplasmic 1 fragment of actin (spot **3**, ↑2.5-fold) and *neuronal plasticity*: β-synuclein (spot **1**, ↓2.0-fold).

**Table 2:** Subcellular localization and function of morphine-altered proteins in hippocampus of experimental groups (+M10) and (−M10) of rats.

Spot	Accession number	Protein name	Change (fold)	Subcellular localization	Molecular functions and biological processes
**1**	SYUB_RAT	Beta-synuclein	↓ 2.0	Cytoplasm	Alpha/beta tubulin-binding, neuronal plasticity
**2**	SYUA_RAT	Alpha-synuclein	↓ 2.5	Cytoplasm, cell membrane, nucleus	Regulation of dopamine release and transport, anti-apoptotic protein
**3**	ACTB_RAT	Actin, cytoplasmic 1 (fragment)	↑ 2.5	Cytoplasm, cytoskeleton	ATP binding, cell motility
**4,5**	ENOA_RAT	Alpha-enolase	↑ 2.2, ↑ 2.2	Cytoplasm, cell membrane	Glycolysis, plasminogen activation, heat-shock protein (HSP48)
**6**	DLDH_RAT	Dihydrolipoyl dehydrogenase, mitochondrial	↑ 2.0	Mitochondrion matrix, cytoplasmic vesicle	Krebs cycle, glycolysis, aging
**7,8**	D3ZGY4_RAT	Glyceraldehyde-3-phosphate dehydrogenase	↓ 2.3, ↓ 2.3	Cytoplasm, nucleus	Glycolysis, apoptosis, oxidative stress

### Comparative proteomic analysis of rat hippocampus from animals exposed to morphine for 10 days and sacrificed 20 days since the last dose of morphine (+M10/−M20) and from control animals (−M10/−M20) by 2D-DIGE and MALDI-TOF MS/MS

**Fig 4:**
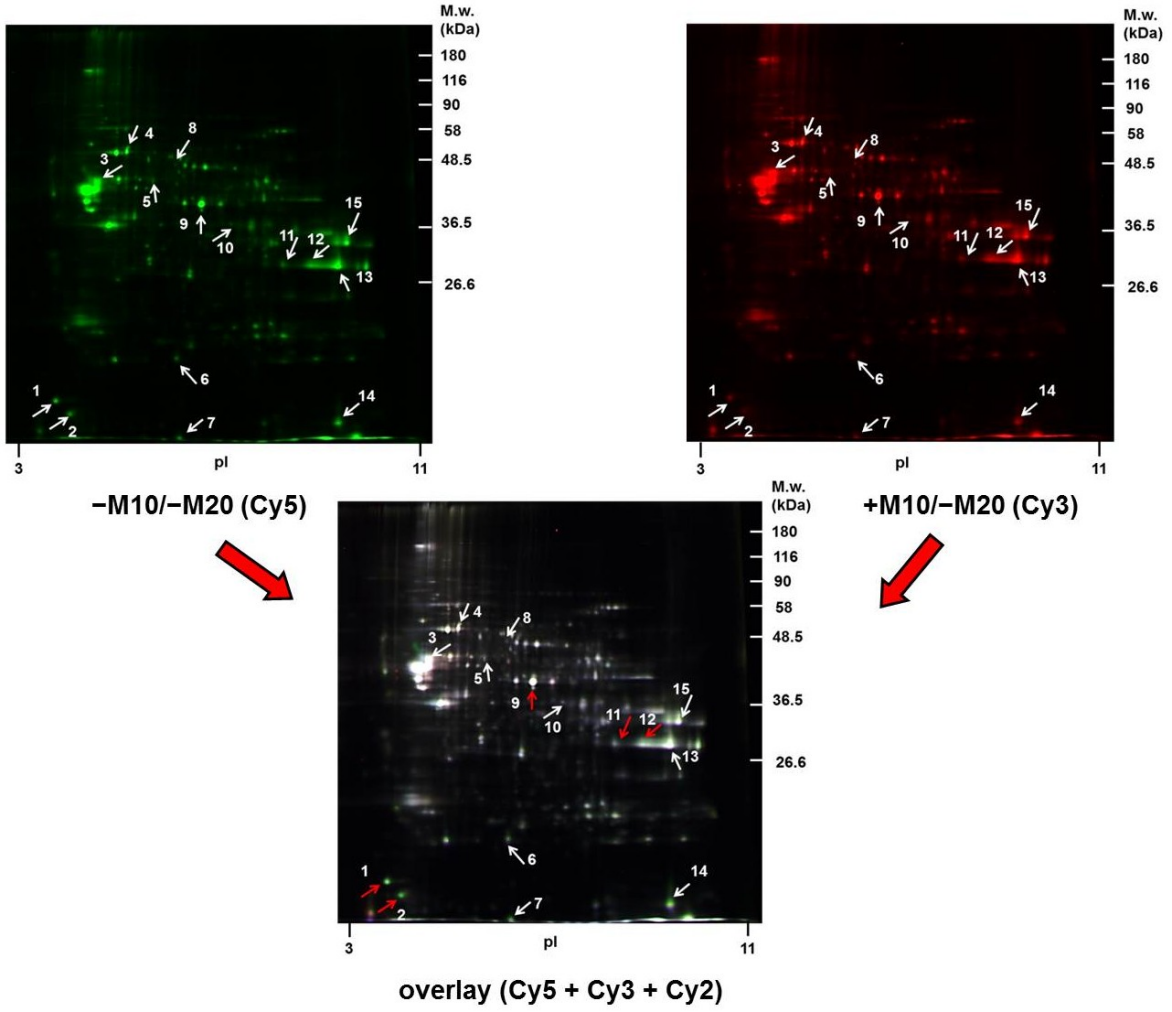
The 2D-DIGE comparative proteomic analysis of PNS fractions prepared from rat hippocampus of control (−M10/−M20) and morphine-treated rats (+M10/−M20) exposed to increasing doses of morphine for 10 days and subsequently nurtured for 20 days without this drug. The PNS samples were prepared as described in Methods and separated in the first dimension on pH 3–11 IPG strips and then by SDS-PADE on 10% acrylamide gels. The analysis of DIGE gels was done by PDQuest^™^. Protein spots (1–15) showing quantitatively significant difference at least 2-fold (p≤0.05) were excised from the CBB-stained gels and identified by MALDI-TOF MS/MS (white + red arrows). The red arrows indicate those protein spots which were altered in (±M10) as well as (±M10/−M20) samples of PNS: 1, 2, 9, 11 and 12. The positions of molecular weight markers are indicated on the right side and pI at the bottom of each gel.

Samples of PNS were labeled with Cy5 (−M10/−M20) or Cy3 (+M10/−M20) and resolved by 2D-ELFO as described in Methods together with internal standard labeled with Cy2 (Fig 4). The total number of protein spots detected by PDQuest^™^ (Bio-Rad) was 343. Only spots showing at least 2-fold quantitatively significant difference (p≤0.05) were excised from the CBB-stained gels (Fig 2B, lower panels), digested with trypsin and used for mass spectrometric analysis. Analysis by MALDI-TOF MS/MS identified 15 altered protein spots (Table 3). As the same protein was identified in spots **11, 12, 13** (glyceraldehyde-3-phosphate dehydrogenase), the number of morphine-altered proteins was 13. A complete list of peptides used for recognition of these proteins is presented in S2 Table.

**Table 3:** MALDI-TOF MS/MS analysis of fifteen altered protein spots in PNS prepared from hippocampus of rats exposed to morphine for 10 days and sacrificed 20 days after the last dose; *difference of protein composition in PNS samples prepared from groups (+M10/−M20) and (*−*M10/−M20)*.

Spot	Accession number	Protein name	Mascot score	Matched peptides	SC^a^ [%]	MW^b^ (kDa)	pI^c^	Change (fold)	p value
**1**	SYUB_RAT	Beta-synuclein	96	5	29	14.5	4.48	↓ 2.4	0.0259
**2**	SYUA_RAT	Alpha-synuclein	249	5	36	14.5	4.74	↓ 5.3	0.0038
**3**	TBA1A_RAT	Tubulin alpha-1A chain	298	19	40	50.8	4.94	↓ 4.5	0.0023
**4**	F1M953_RAT	Stress-70 protein, mitochondrial	423	15	15	74.0	5.87	↑ 2.1	0.0291
**5**	PDIA3_RAT	Protein disulfide-isomerase A3	117	10	13	57.0	5.88	↓ 2.2	0.0286
**6**	ATP5H_RAT	ATP synthase subunit d, mitochondrial	305	8	31	18.8	6.17	↓ 2.0	0.0167
**7**	SODC_RAT	Superoxide dismutase [Cu-Zn]	81	6	31	16.1	5.88	↓ 3.1	0.0490
**8**	DPYL2_RAT	Dihydropyrimidinase-related protein 2	237	9	17	62.6	5.95	↓ 2.4	0.0251
**9**	ENOA_RAT	Alpha-enolase	694	20	28	47.4	6.16	↑ 3.1	0.0154
**10**	EFTU_RAT	Elongation factor Tu, mitochondrial	242	8	13	49.9	7.23	↓ 2.2	0.0164
**11**	D3ZGY4_RAT	Glyceraldehyde-3-phosphate dehydrogenase	109	9	9	36.1	7.63	↓ 2.4	0.0168
**12**	D3ZGY4_RAT	Glyceraldehyde-3-phosphate dehydrogenase	273	6	10	36.1	7.63	↓ 2.3	0.0087
**13**	D3ZGY4_RAT	Glyceraldehyde-3-phosphate dehydrogenase	316	13	20	36.1	7.63	↓ 4.1	0.0180
**14**	COF1_RAT	Cofilin-1	346	9	40	18.7	8.22	↓ 2.8	0.0115
**15**	ALDOA_RAT	Fructose-bisphosphate aldolase A	255	24	53	39.8	8.31	↓ 2.8	0.0104

^*a*^ sequence coverage,

^*b*^ theoretical molecular weight,

^*c*^ theoretical isoelectric point; complete list of peptides used for identification is presented in S2 Table.

When compared with hippocampus prepared from (±M10) groups of rats (Fig 1 and Table 1), the number of altered protein spots was increased from *eight* to *fifteen* (Fig 4 and Table 3). Noticeably, the alteration of *five* protein spots identified in (±M10) samples of PNS: β-synuclein (spot **1**), α-synuclein (spot **2**), alpha-enolase (spot **5**) and glyceraldehyde-3-phosphate dehydrogenase (spots **7, 8**) persisted for 20 days since the last addition of the drug: α-synuclein (spot **1**), β-synuclein (spot **2**), α-enolase (spot **9**) and glyceraldehyde-3-phosphate dehydrogenase (spots **11, 12** and **13**).

**Fig 5:**
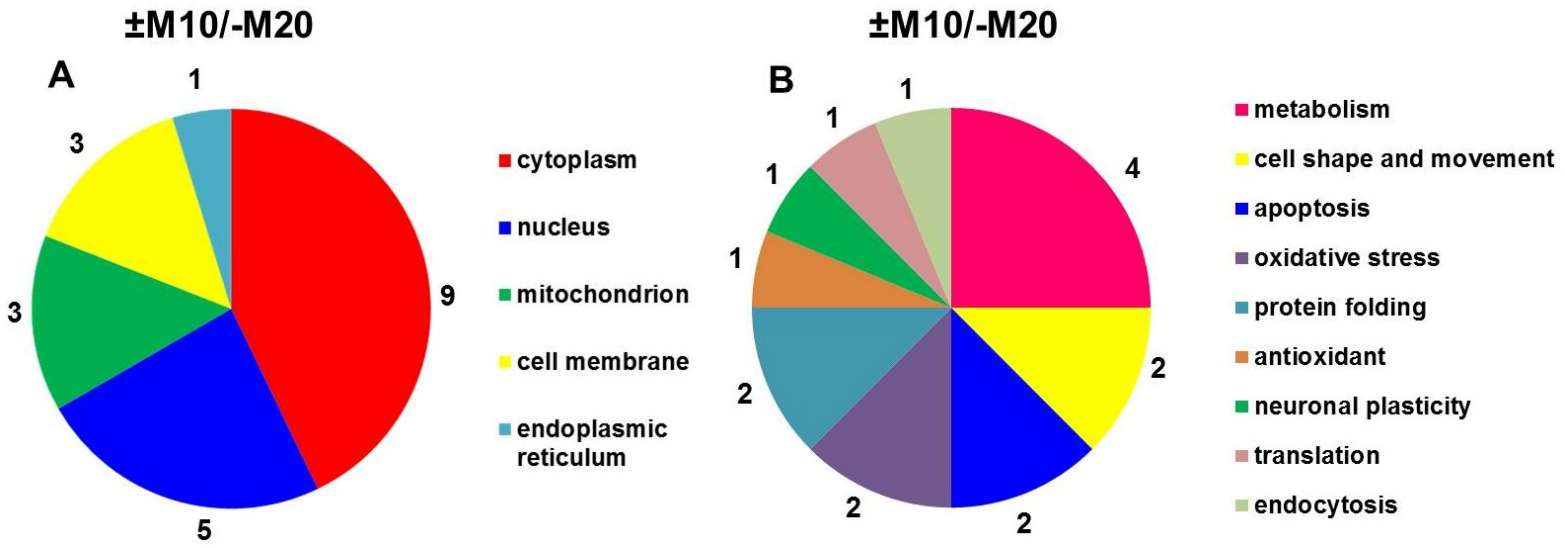
Subcellular localization (A) and functional significance (B) of morphine-altered proteins in experimental groups (+M10/−M20) and (−M10/−M20) identified by MALDI-TOF MS/MS.

Protein categorization (subcellular localization and functional significance) performed according to the current annotations in the UniProt database (Table 4, Fig 5) distinguished proteins functionally involved in *glycolysis*, *oxidative stress* and *apoptosis*: glyceraldehyde-3-phosphate dehydrogenase (↓2.4-fold; ↓2.3-fold; ↓4.1-fold, spots **11, 12, 13**); *glycolysis*: α-enolase (↑3.1-fold, spot **9**); *oxidative phosphorylation*: mitochondrial ATPase synthase subunit d (↓2.0-fold, spot **6**); *cell shape and movement*: tubulin α-1A chain (↓4.5-fold, spot **3**), cofilin-1 (↓2.8-fold, spot **14**); *apoptosis*: α-synuclein (↓5.3-fold, spot **2**), *oxidative stress*: dihydropyrimidinase-related protein 2 (↓2.4-fold, spot **8**), *protein folding*: mitochondrial stress-70 protein (↑2.1-fold, spot **4**), protein disulfide-isomerase A3 (↓2.2-fold, spot **5**); *detoxification of reactive oxygen species*: superoxide dismutase [Cu-Zn] (↓3.1-fold, spot **7**); *neuronal plasticity*: β-synuclein (↓2.4, **spot 1**); *translation*: mitochondrial elongation factor Tu (↓2.2-fold, spot **10**), and *endocytosis*: dihydropyrimidinase-related protein 2 (↓2.4-fold, spot **8**).

**Table 4:** Subcellular localization and function of morphine-altered proteins in hippocampus of experimental groups (+M10/−M20) and (−M10/−M20) of rats.

Spot	Accession number	Protein name	Change (fold)	Subcellular localization	Molecular functions and biological processes
**1**	SYUB_RAT	Beta-synuclein	↓ 2.4	Cytoplasm	Alpha/beta tubulin-binding, neuronal plasticity
**2**	SYUA_RAT	Alpha-synuclein	↓ 5.3	Cytoplasm, cell membrane, nucleus	Regulation of dopamine release and transport, anti-apoptotic protein
**3**	TBA1A_RAT	Tubulin alpha-1A chain	↓ 4.5	Cytoplasm, cytoskeleton	GTP binding, microtubule-based process, cell division
**4**	F1M953_RAT	Stress-70 protein, mitochondrial	↑ 2.1	Mitochondrion, nucleus	Protein folding, anti-apoptotic protein, erythrocyte differentiation
**5**	PDIA3_RAT	Protein disulfide-isomerase A3	↓ 2.2	Endoplasmic reticulum, melanosome	Cell redox homeostasis, protein folding, neurodegenerative processes
**6**	ATP5H_RAT	ATP synthase subunit d, mitochondrial	↓ 2.0	Mitochondrion	Oxidative phosphorylation, hydrogen ion transport
**7**	SODC_RAT	Superoxide dismutase [Cu-Zn]	↓ 3.1	Cytoplasm, nucleus	Antioxidant, detoxification of reactive oxygen species
**8**	DPYL2_RAT	Dihydropyrimidinase-related protein 2	↓ 2.4	Cytoplasm, cytoskeleton, cell membrane	Neurogenesis, endocytosis, control of oxidative stress
**9**	ENOA_RAT	Alpha-enolase	↑ 3.1	Cytoplasm, cell membrane	Glycolysis, plasminogen activation, heat-shock protein (HSP48)
**10**	EFTU_RAT	Elongation factor Tu, mitochondrial	↓ 2.2	Mitochondrion	Elongation factor, translation
**11,12,13**	D3ZGY4_RAT	Glyceraldehyde-3-phosphate dehydrogenase	↓ 2.4, ↓ 2.3, ↓ 4.1	Cytoplasm, nucleus	Glycolysis, apoptosis, oxidative stress
**14**	COF1_RAT	Cofilin-1	↓ 2.8	Cytoplasm, cytoskeleton, nucleus	Actin cytoskeleton, hippocampus development
**15**	ALDOA_RAT	Fructose-bisphosphate aldolase A	↓ 2.8	Cytoplasm	Glycolysis, gluconeogenesis

### Immunoblot analysis of α- and β-synuclein, GAPDH and actin in rat hippocampus of experimental groups (±M10) and (±M10/−M20)

In order to verify the results obtained by comparative 2D-DIGE, PNS hippocampal fractions prepared from groups (−M10), (+M10), (+M10/−M20) and (−M10/−M20) were resolved by 2D-ELFO and the content of α- and β-synuclein, glyceraldehyde-3-phosphate dehydrogenase (GAPDH) and actin was determined by immunoblotting with specific antibodies.

**Fig 6:**
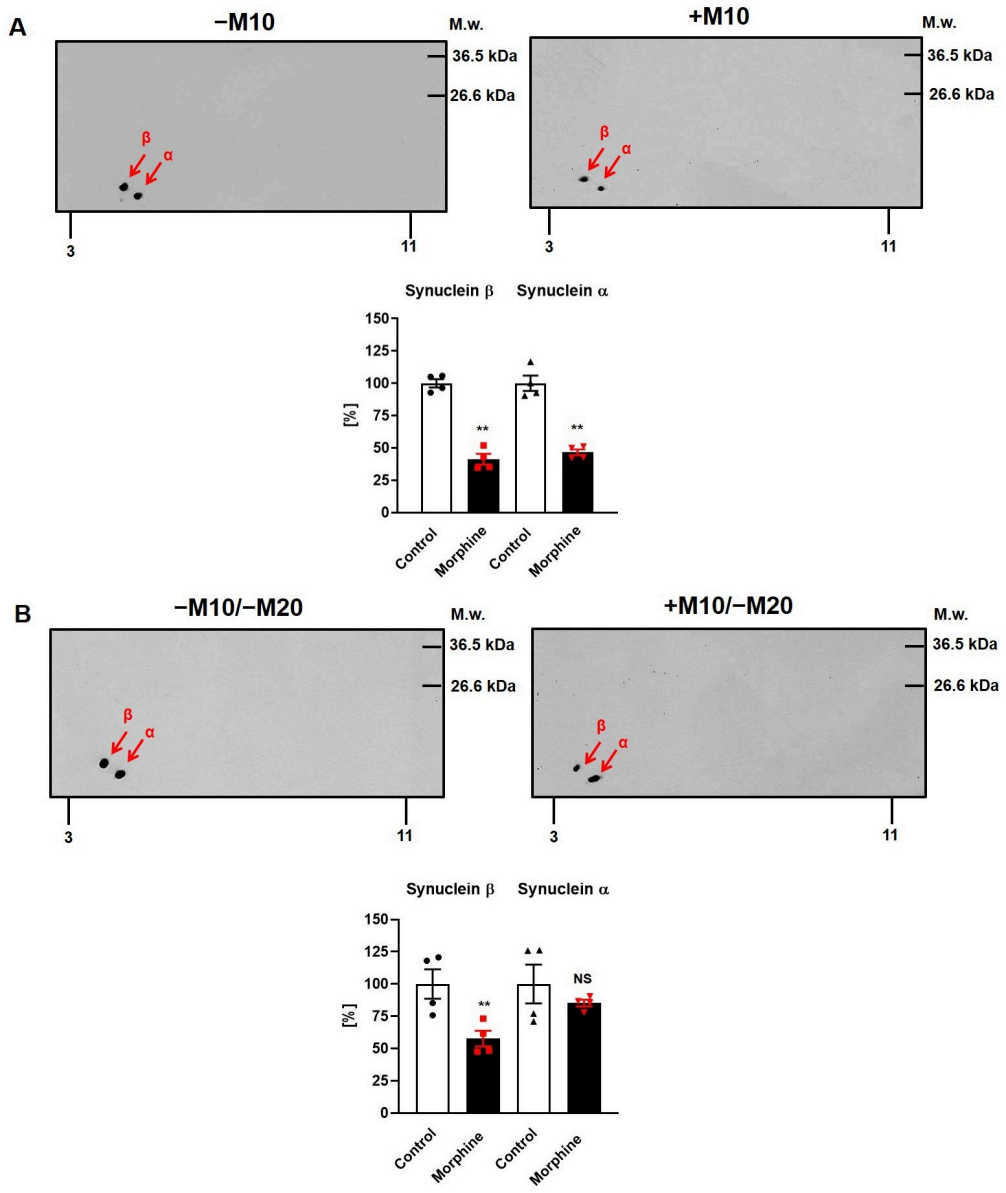
2D immunoblot analysis of α/β-synucleins in PNS prepared from rat hippocampus of experimental groups (±M10) and (±M10/−M20). **(A)** The (+M10) and (−M10) or **(B)** (+M10/−M20) and (−M10/−M20) samples of PNS (2000 μg protein per gel) were extracted in acetone/TCA and resolved by 2D-ELFO as described in Methods. The α- and β-synucleins were recognized by Ab F-11 (sc-514908). The two immunoblot signals with similar M_w_ of ≈ 14–15 kDa were observed at pI ≈ 4.3 and ≈ 4.7. In group (+M10) (**A**, upper right), the level of β-synuclein was decreased to 41.3% (p<0.01) when compared with control group (−M10) (**A**, upper left). The α-synuclein was decreased to 46.6% (p<0.01). In group (+M10/−M20) (**B**, lower right), a significant down-regulation of β-synuclein (58%, p<0.01) and down-regulation of α-synuclein (85%, p>0.05) was observed as well when compared with intensities of immunoblot signals of these two proteins in group (−M10/−M20) (**B**, lower left). Data present bar graphs with the individual data points along with the bar with error. The significance of the difference between the (±M10) or (±M10/−M20) samples of PNS was analyzed by Student´s *t*-test using GraphPad*Prism4*; *, p<0.05; **, p<0.01; NS, p>0.05.

In (±M10) samples of PNS, position of immunoblot signals of α- and β-synuclein in 2D gels (Fig 6A) was the same as in 2D-DIGE gels [compare with Fig 1 and Table 1; spot **1** (β-synuclein, ↓2.0-fold) and spot **2** (α-synuclein, ↓2.5-fold)]. The M_w_ of these two proteins was ≈ 14 and 16 kDa, and their pI values were ≈ 4.3 and ≈ 4.7, respectively. Intensities of immunoblot signals were decreased to 41.3% (β-synuclein, p<0.01) and 46.6% (α-synuclein, p<0.01) when compared with corresponding controls. Thus, results obtained by 2D-DIGE indicating the decrease of these two proteins by morphine-treatment, were verified by immunoblot analysis of 2D gels. The difference in the magnitude of the decrease may be attributed to the major difference in methodology of detection combined with the necessity to apply largely different amounts of total protein per gel (75 μg versus 2000 μg) to achieve the proper labeling of the sample with Cy probes on the one hand and intensity of immunoblot signals on the other hand [26,27].

Accordingly, the densitometric scanning of immunoblot signals of α/β-synucleins in (±M10/−M20) samples of PNS indicated a significant down-regulation to 58% (β-synuclein, p<0.01) and 85% (α-synuclein, p>0.05), respectively (Fig 6B). When combined together, the results of both 2D-DIGE and immunoblot analyses indicated consistently the down-regulation of α- and β-synuclein in hippocampus of rats exposed to morphine for 10 days. *Noticeably*, *this down-regulation persisted for 20 days of abstinence*.

**Fig 7:**
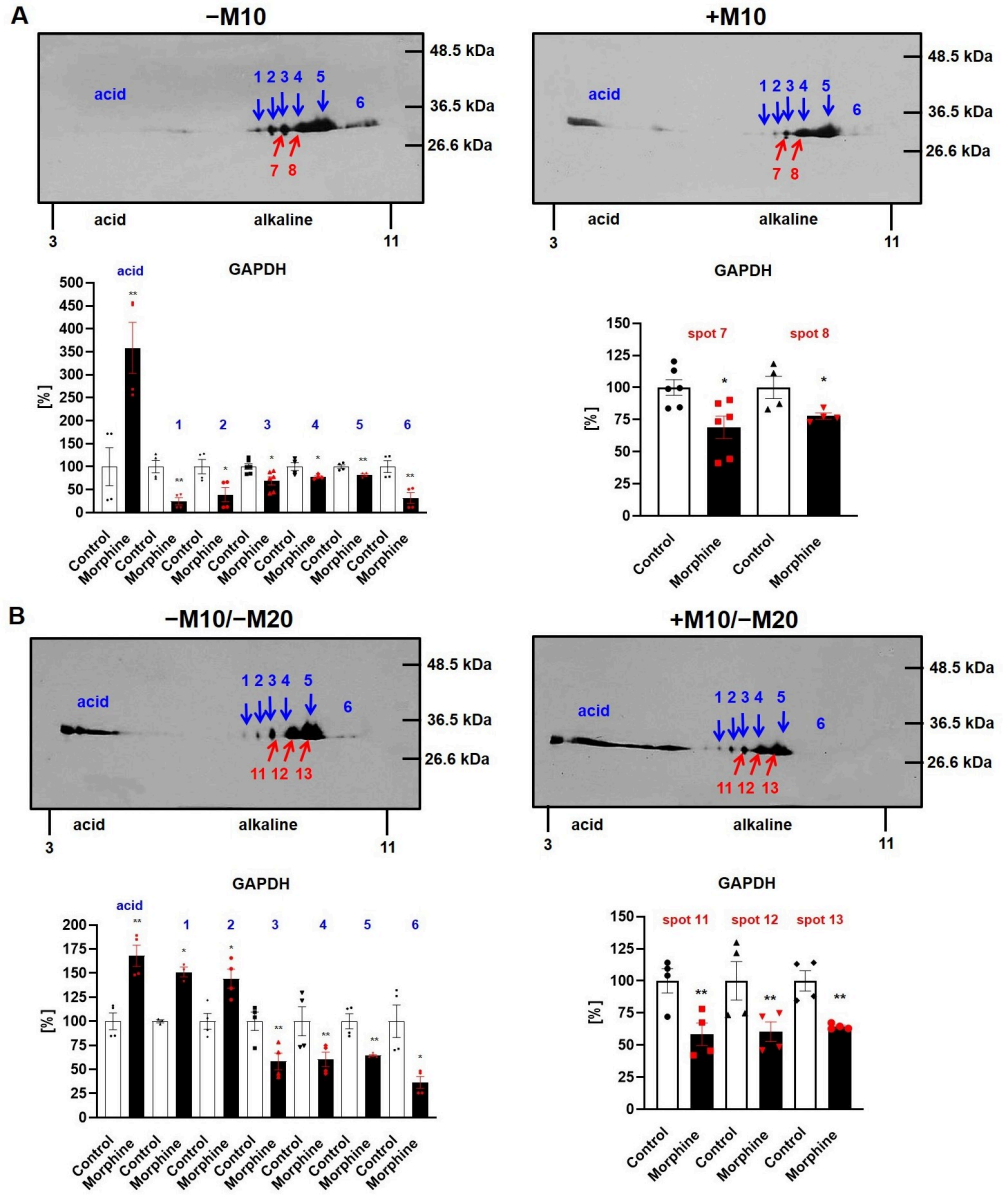
2D immunoblot analysis of GAPDH in PNS prepared from rat hippocampus of experimental groups (±M10) and (±M10/−M20). **(A)** The (+M10) and (−M10) or **(B)** (+M10/−M20) and (−M10/−M20) samples of PNS (2000 μg protein per gel) were extracted in acetone/TCA, resolved by 2D-ELFO and immunobloted with Ab FL-335 (sc-25778) as described in Methods. The seven immunoblot signals (acid + 1–6) exhibited the M_w_ ≈ 36 kDa were distributed over a wide range of pI (3.5–10) in both groups of experimental animals. In (**A**), the immunoblot signals 3 and 4 exhibited the same pI as spots 7 and 8 which were recognized as significantly decreased (≥2-fold) by DIGE analysis (compare with Figs 1 and 2A and Table 1). In (**B**), the immunoblot signals 3, 4 and 5 were identical with spots 11, 12 and 13, which were recognized as significantly decreased (≥2-fold) by DIGE analysis (compare with Figs 4 and 2B and Table 3). Data present bar graphs with the individual data points along with the bar with error. The significance of the difference between the (±M10) or (±M10/−M20) samples of PNS was analyzed by Student´s *t*-test using GraphPad*Prism4*; *, p<0.05; **, p<0.01.

The immunoblot signals of GAPDH detected by Ab FL-335 were distributed over a wide range of pI ≈ 3.5–10, but the M_w_ ≈ 36 kDa of all these signals was the same in PNS prepared from all four groups of experimental animals, Fig 7. Morphine-treatment resulted in redistribution of GAPDH signals between alkaline and acid region of the gel manifested as a transfer from alkaline to acid region (Fig 7A, upper panels). Consequently, intensities of individual spots in alkaline region (blue arrows) were decreased in (+M10) when compared with (−M10) samples of PNS to: ≈ 25% (spot 1, p<0.01), ≈ 39% (spot 2, p<0.05), ≈ 69% (spot 3, p<0.05), ≈ 78% (spot 4, p<0.05), ≈ 82% (spot 5, p<0.01), and ≈ 32% (spot 6, p<0.01), respectively. Spots **3** and **4** were those identified in DIGE gels as spots **7** and **8** (red arrows; compare with Figs 1 and 2A and Table 1). The signal detected in the acid region was increased to 358% (p<0.01). The sum of signals in alkaline region (1+2+3+4+5+6) was decreased to 67% (p<0.05); the total signal of all spots was decreased to 72% (p<0.05).

Similar results were obtained in (±M10/−M20) samples of PNS. GAPDH signals were distributed over a wide range of pI ≈ 3.5–10, M_w_ of all isoforms of this enzyme was ≈ 36 kDa and morphine-treatment resulted in the transfer from alkaline to acid region of the gel. However, the relative magnitude of this transfer was less than in (±M10) samples of PNS (Fig 7B, lower panels). Intensities of individual spots in alkaline region (blue arrows) were decreased to ≈ 58% (spot 3, p<0.01), ≈ 60% (spot 4, p<0.01), ≈ 64% (spot 5, p<0.01) and ≈ 36% (spot 6, p<0.05); the two minor spots (1 and 2) were increased to ≈ 151% (p<0.05) and ≈ 145% (spot 2, p<0.05), respectively. The major spots **3**, **4** and **5** were those identified in DIGE gels as spots **11**, **12** and **13** (red arrows; please compare with Figs 4 and 2B and Table 3). The total signal of GAPDH subunits in alkaline region (1+2+3+4+5+6) was decreased to 72% (p<0.05); the total signal of all spots was unchanged by morphine (NS, p>0.05).

**Fig 8:**
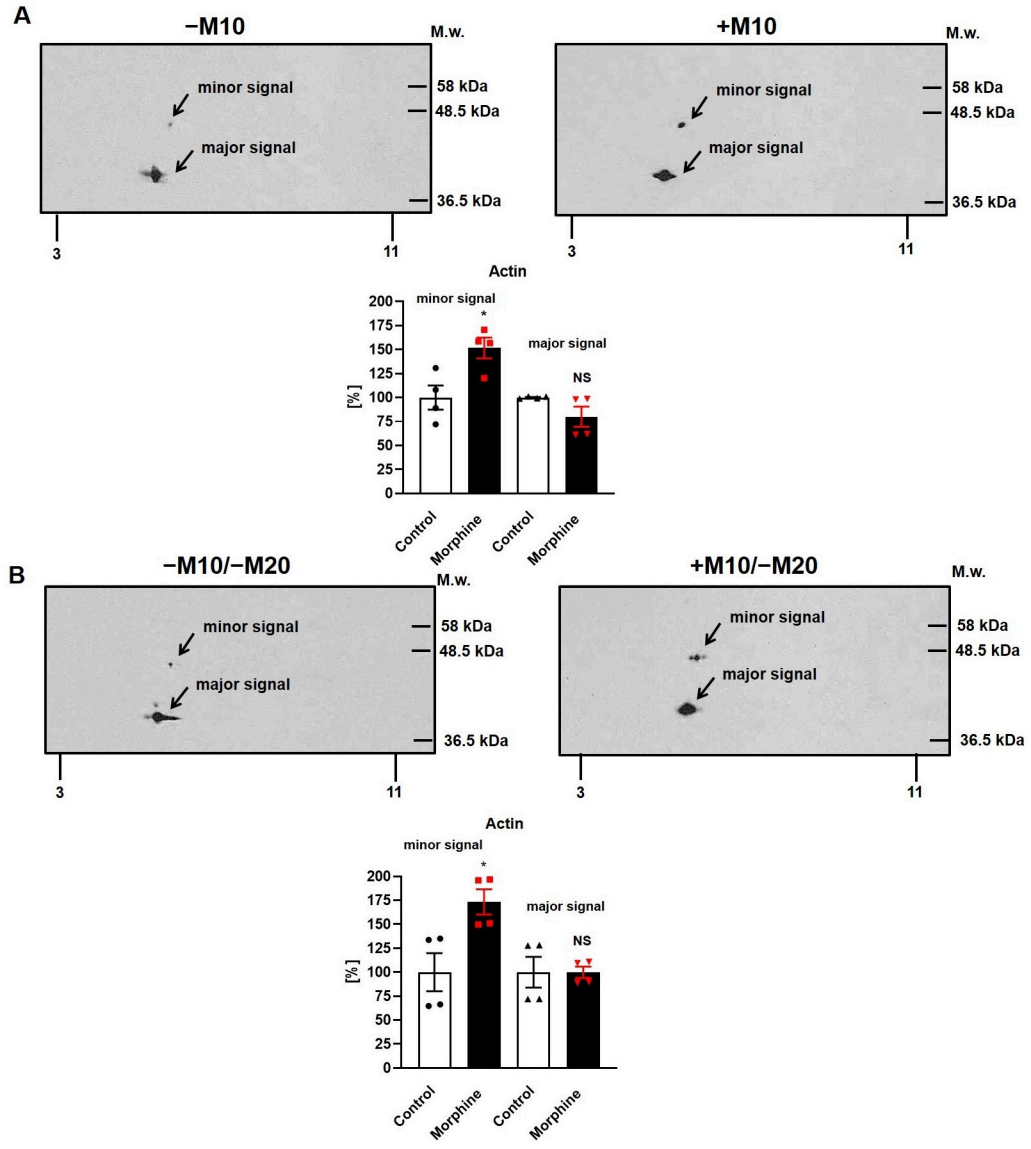
2D immunoblot analysis of actin in PNS prepared from rat hippocampus of experimental groups (±M10) and (±M10/−M20). The (+M10) and (−M10) **(A)** or (+M10/−M20) and (−M10/−M20) **(B)** samples of PNS (200 μg protein per gel) were extracted in acetone/TCA, resolved by 2D-ELFO and immunoblotted as described in Methods. The two signals were recognized with Ab I-19 (sc-1616): the major signal with M_w_ ≈ 42 kDa at pI of ≈ 5.2–5.8 and the minor signal with M_w_ ≈ 46 kDa at pI of ≈ 5.4. Data present bar graphs with the individual data points along with the bar with error. The significance of the difference between (±M10) or (±M10/−M20) samples of PNS was analyzed by Student´s *t*-test using GraphPad*Prism4*; *, p<0.05; NS, p>0.05.

The total signal of actin was unchanged, Fig 8. In (±M10) samples of PNS (Fig 8A), the major signal of actin was decreased to 80%, but this decrease was not significant when averaged in three immunoblots (NS, p>0.05); the minor signal was increased to 152% (*, p<0.05). The total signal of both spots was unchanged (NS, p>0.05). The same result was obtained in (±M10/−M20) groups of rats (Fig 8B): the major signal of actin was unchanged when averaged in three blots (NS, p>0.05), the minor signal was increased to 173% (*, p<0.05). The total signal of both spots was unchanged (NS, p>0.05). As actin is known to be modified by sugars, the increased level of its minor fragment exhibiting the higher molecular weight may represent the glycosylated form detected in both (±M10) and (±M10/−M20) samples of hippocampus.

The level of beta-actin commonly used as a loading control was also detected and was unchanged (NS, p>0.05), S1 File. Moreover, 1D immunoblot resolution of GAPDH was performed (S2 File). The difference in the expression level between (±M10) and (±M10/−M20) samples was not significant.

### Label-free MS analysis of rat hippocampus from animals exposed to morphine for 10 days (±M10); comparison with animals exposed to morphine and subsequently nurtured for 20 days in the absence of this drug (±M10/−M20)

**Fig 9:**
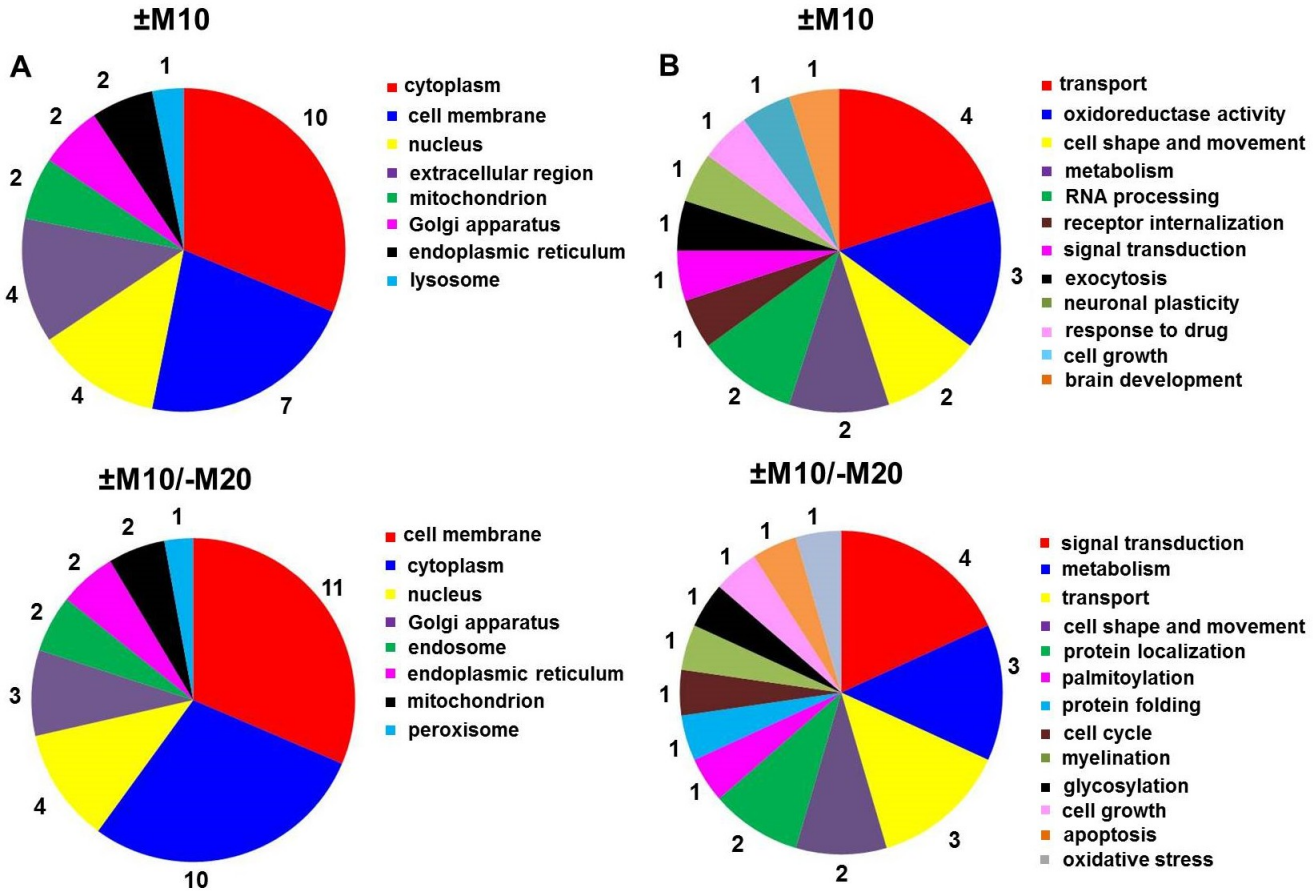
Subcellular localization (A) and functional significance (B) of morphine-altered proteins in experimental groups ±M10 (upper panels) and ±M10/−M20 (lower panels) identified by MaxLFQ.

In the last part of our work, we applied the gel-free, label-free proteomic approach denominated as MaxLFQ [21]. The LFQ analysis identified 19 proteins with significantly altered expression level (≥1.7-fold) in group (+M10) when compared with group (−M10), Table 5, Fig 9, upper panels. According to the current annotations in the UniProt database, the identified proteins were functionally related to *transport* (**1**-solute carrier family 22 member 23, 4-urea transporter 1, **17**-GTP-binding protein SAR1b, **18**-peflin), *oxidoreductase activity* (**2**-ECSIT, **6**-ATP synthase subunit f, **19**-NADPH-dependent diflavin oxidoreductase 1), *cell shape and movement* (**8**-filamin A, **11**-plastin 1), *metabolism* (**10**-beta-galactosidase, **12**-GMP reductase), *RNA processing* (**15**-alanyl-tRNA editing protein Aarsd1, **16**-RNA polymerase II-associated protein 3), *receptor internalization* and *brain development* (**3**-CD9 antigen), *signal transduction* (**5**-SH2B adapter protein 1), *exocytosis* (**7**-exocyst complex component 1), *neuronal plasticity* (**9**-pro-MCH), *response to drug* (**13**-protein phosphatase 1E), and *cell growth* (**14**-alpha-2-HS-glycoprotein).

**Table 5:** Subcellular localization and function of morphine-altered proteins in groups (+M10) and (−M10) identified by MaxLFQ.

Accession number	Protein name	Change (fold)	p value	Subcellular localization	Molecular functions and biological processes
**1**-Q9QZG1	Solute carrier family 22 member 23	↑ 3.1	0.0001	Cell membrane	Membrane integrated transporter, ion transport
**2**-Q5XIC2	Evolutionarily conserved signaling intermediate in Toll pathway, mitochondrial	↑ 3.0	0.0012	Mitochondrion, nucleus, cytoplasm	Assembly of mitochondrial NADH
**3**-P40241	CD9 antigen	↑ 2.7	0.0159	Cell membrane, extracellular region	Brain development, cell adhesion, receptor internalization
**4**-P97689	Urea transporter 1	↑ 2.2	0.0127	Cell membrane	Urea transport
**5**-Q62985	SH2B adapter protein 1	↑ 2.2	0.0439	Nucleus, cytoplasm, cell membrane	Signal transduction, tyrosine kinase adaptor activity
**6**-D3ZAF6	ATP synthase subunit f, mitochondrial	↑ 1.7	0.0363	Mitochondrion	Hydrogen ion transport, cristae formation
**7**-F1LQN9	Exocyst complex component 1	↑ 1.7	0.0034	Cell membrane	Exocytosis
**8**-C0JPT7	Filamin A	↓ 3.8	0.0223	Cytoplasm—cytoskeleton, nucleus, cell membrane	Actin cytoskeleton reorganization
**9**-P14200	Pro-MCH	↓ 2.3	0.0002	Extracellular region	Regulation of synaptic plasticity, glucose homeostasis
**10**-D3ZUM4	Beta-galactosidase	↓ 2.0	0.0200	Golgi apparatus, lysosome, extracellular region	Galactose catabolism
**11**-A0A0G2QC04	Plastin 1	↓ 1.9	0.0231	Cytoplasm—cytoskeleton	Actin cytoskeleton reorganization
**12**-Q5FVP6	GMP reductase	↓ 1.9	0.0002	Cytoplasm	Purine metabolism
**13**-Q80Z30	Protein phosphatase 1E	↓ 1.9	0.0062	Nucleus, cytoplasm	Negative regulation of protein kinase activity, response to drug, positive regulation of stress fiber assembly
**14**-P24090	Alpha-2-HS-glycoprotein	↓ 1.9	0.0005	Extracellular region	Organ regeneration, negative regulation of cell growth
**15**-Q5XI97	Alanyl-tRNA editing protein Aarsd1	↓ 1.8	0.0005	Cytoplasm	RNA processing
**16**-Q68FQ7	RNA polymerase II-associated protein 3	↓ 1.8	0.0094	Cytoplasm	RNA processing
**17**-Q5HZY2	GTP-binding protein SAR1b	↓ 1.7	0.0044	Endoplasmic reticulum, Golgi apparatus	Protein transport, vesicle organization
**18**-Q641Z8	Peflin	↓ 1.7	0.0065	Endoplasmic reticulum, cytoplasm, cell membrane	Response to calcium ion, ER to Golgi vesicle-mediated transport
**19**-D4ABT4	NADPH-dependent diflavin oxidoreductase 1	↓ 1.7	0.0002	Cytoplasm	Electron transfer chain

The LFQ analysis of proteins in PNS prepared from rats sacrificed 20 days since the last dose of morphine (group +M10/−M20) identified 20 proteins with significantly altered expression level (≥1.7-fold) when compared with group (−M10/−M20), Table 6, Fig 9, lower panels. Those 20 proteins were functionally related to *signal transduction* (**10**-rho GTPase-activating protein 39, ↑1.7-fold; **15**-crk-like protein, ↓1.9-fold; **17**-SH2B adapter protein 1, ↓1.9-fold; **19**-regulator of G-protein signaling 14, ↓1.7-fold), *metabolism* (**1**-aldose 1-epimerase, ↑3.6-fold; **6**-citrate lyase subunit beta-like protein, ↑2.0-fold; **18**-farnesyl pyrophosphate synthase ↓1.8-fold), *transport* (**2**-equilibrative nucleoside transporter 2, ↑3.1-fold; **9**-ADP/ATP translocase 2, ↑1.7-fold; **16**-AP complex subunit sigma, ↓1.9-fold), *cell shape and movement* (**11**-signal-induced proliferation-associated 1-like protein 1, ↑1.7-fold; **7**-transmembrane protein 35A, ↑1.8-fold), *protein localization* (**8**-A-kinase anchor protein 7 isoforms delta and gamma, ↑1.7-fold; **13**-Golgin B1, ↓2.4), *palmitoylation* (**3**-alpha/beta hydrolase domain-containing protein 17B, ↑2.7-fold), *protein folding* (**4**-alpha-2-macroglobulin receptor-associated protein, ↑2.2-fold), *regulation of cell cycle* (**5**-calcium/calmodulin-dependent protein kinase II inhibitor 1, ↑2.1-fold), *myelination* (**12**-neurofascin, ↓3.0-fold), *glycosylation* (**14**-protein O-linked-mannose beta-1,4-N-acetylglucosaminyltransferase 2, ↓2.2-fold), *apoptosis* and *oxidative stress* (**20**-alpha-synuclein, ↓1.7-fold).

**Table 6:** Subcellular localization and function of morphine-altered proteins in groups (+M10/−M20) and (−M10/−M20) identified by MaxLFQ.

Accession number	Protein name	Change (fold)	p value	Subcellular localization	Molecular functions and biological processes
**1**-Q66HG4	Aldose 1-epimerase	↑ 3.6	0.0073	Cytoplasm	Carbohydrate metabolism
**2**-O54699	Equilibrative nucleoside transporter 2	↑ 3.1	0.0188	Cell membrane	Nucleoside transport
**3**-Q6AY17	Alpha/beta hydrolase domain-containing protein 17B	↑ 2.7	0.0233	Cell membrane, endosome	Protein palmitoylation/depalmitoylation
**4**-Q99068	Alpha-2-macroglobulin receptor-associated protein	↑ 2.2	0.0140	Endoplasmic reticulum, endosome, Golgi apparatus	Chaperone for LDL-receptor related proteins
**5**-Q9JI15	Calcium/calmodulin-dependent protein kinase II inhibitor 1	↑ 2.1	0.0306	Cell membrane	Negative regulation of cell cycle, negative regulation of proteolysis
**6**-Q5I0K3	Citrate lyase subunit beta-like protein, mitochondrial	↑ 2.0	0.0214	Mitochondrion	Vitamin B12 metabolism
**7**-Q6JAM9	Transmembrane protein 35A	↑ 1.8	0.0430	Peroxisome	Modulation of sympathetic neurite outgrowth
**8**-Q6JP77	A-kinase anchor protein 7 isoforms delta and gamma	↑ 1.7	0.0017	Nucleus, cytoplasm, cell membrane	Protein localization
**9**-Q09073	ADP/ATP translocase 2	↑ 1.7	0.0042	Mitochondrion	ATP:ADP antiporter activity, chromosome segregation
**10**-P18890	Rho GTPase-activating protein 39	↑ 1.7	0.0042	Cytoplasm	Signal transduction
**11**-O35412	Signal-induced proliferation-associated 1-like protein 1	↑ 1.7	0.0008	Cell membrane, cytoplasm	Actin cytoskeleton reorganization, regulation of axonogenesis
**12**-D3ZW56	Neurofascin	↓ 3.0	0.0057	Cell membrane	Myelination, axon guidance
**13**-A0A0G2JWG6	Golgin B1	↓ 2.4	0.0234	Golgi apparatus	Protein localization
**14**-Q5NDF0	Protein O-linked-mannose beta-1,4—N-acetylglucosaminyltransferase 2	↓ 2.2	0.0092	Endoplasmic reticulum	O-linked glycosylation
**15**-Q5U2U2	Crk-like protein	↓ 1.9	0.0174	Cell membrane, cytoplasm	Signal transduction, hippocampus development, regulation of cell growth
**16**-B5DFI3	AP complex subunit sigma	↓ 1.9	0.0188	Cell membrane, cytoplasm, Golgi apparatus	Protein transport
**17**-Q62985	SH2B adapter protein 1	↓ 1.9	0.0099	Nucleus, cytoplasm, cell membrane	Signal transduction, tyrosine kinase adaptor activity
**18**-P05369	Farnesyl pyrophosphate synthase	↓ 1.8	0.0336	Cytoplasm	Lipid metabolism, cholesterol biosynthesis
**19**-O08773	Regulator of G-protein signaling 14	↓ 1.7	0.0033	Cell membrane, cytoplasm, nucleus	Signal transduction, cell division, learning, long-term memory
**20**-P37377	Alpha-synuclein	↓ 1.7	0.0075	Cytoplasm, cell membrane, nucleus	Negative regulation of apoptosis, aging, response to oxidative stress, regulation of dopamine release and transport

## Discussion

### 2D-DIGE and 2D immunoblot analysis of rat hippocampus from animals exposed to morphine for 10 days (±M10); comparison with animals exposed to morphine and subsequently nurtured for 20 days in the absence of this drug (±M10/−M20)

The number of altered proteins in hippocampus was increased from 6 to 13 after 20 days of abstinence. This finding is just the opposite when compared with that observed in forebrain cortex, where the number of differentially expressed proteins was decreased from 28 (±M10) to 14 (±M10/−M20) when determined in CBB-stained 2D gels or from 113 to 19 when determined by LFQ [10]. Noticeably, the altered level of *four* proteins identified in (±M10) samples of PNS (β-synuclein, α-synuclein, alpha-enolase and GAPDH) persisted for 20 days since the withdrawal of morphine (Figs 1 and 4, Tables 1 and 3).

The α-synuclein is a pathological protein functionally related to production of “Lewy bodies” in Parkinson´s disease and dementia [28–31]. While its aggregation represents the major risk factor for neurodegeneration, its function under physiological conditions remains poorly understood. It has been suggested that α-synuclein is involved in negative regulation of dopaminergic neurotransmission, synaptic vesicle cycling, synaptic plasticity and neuroprotection [32]. Interestingly, Ziolkowska et al. [33] observed the down-regulation of α-synuclein mRNA in the basolateral amygdala, dorsal striatum, nucleus accumbens and ventral tegmental area of mice withdrawn from chronic morphine treatment for 48 h; on the other hand, protein level of α-synuclein, determined in the same brain regions, was up-regulated. β-synuclein was described as the non-amyloidogenic homolog of α-synuclein with anti-apoptotic effect [34]. The evidence for down-regulation of α/β synucleins persisting for 20 days since morphine withdrawal was confirmed by 2D immunoblot analysis, Fig 6.

In 2010, the group of Dr. Piotr Suder created the *Morphinome Database* (www.addiction-proteomics.org) in order to facilitate the search for the proteins altered by morphine administration. This database is continuously updated. More than 20% of proteins distinguished in this database are functionally related to regulation of energy metabolism—glycolysis, gluconeogenesis, Krebs cycle and oxidative phosphorylation [35].

In our study, the down-regulation of glycolytic enzyme GAPDH was observed after 10 days of morphine treatment and this decrease was also noticed in hippocampus of rats sacrificed after 20 days of drug withdrawal. Noticeably, the 2D immunoblot analysis recognized the subset of six GAPDH spots (in both ±M10 and ±M10/−M20 samples of PNS) which were present in alkaline region of 2D gels and selectively decreased by morphine (Fig 7). As GAPDH was found to be extensively modified by post-translational modifications [36–40], and post-translational modifications of this enzyme were suggested to play a role in oxidative stress and apoptosis [41,42], it is reasonable to assume that morphine-induced change of six GAPDH subunits reveals different functions fulfilled by individual variants of this enzyme.

One of the functional consequences of manifestation of oxidative stress is the decrease of cellular ATP level and blockade of glycolysis [43]. Accordingly, proteomic analysis of hippocampus from mice exposed to morphine for 10 days indicated a decreased level of three enzymes related to regulation of glycolysis and mitochondria: E2 component of the pyruvate dehydrogenase complex, lactate dehydrogenase 2, and Fe-S protein 1 of NADH dehydrogenase [44]. Further view on literature data dealing with morphine-induced alteration of CNS energy metabolism suggests that chronic morphine treatment impairs the glucose metabolism and ATP production. This effect is associated with morphine withdrawal symptoms and impairment in memory [45].

Our data also indicated an increased level of α-enolase, another glycolytic enzyme participating in a multitude of pathological processes. Increased level of α-enolase was again noticed in (±M10) as well as (±M10/−M20) samples of hippocampus (Tables 1 and 3). Based on comparative proteomic analysis of human, mouse and rat tissues, Díaz-Ramos et al. [46] reported that α-enolase could be regarded as a marker of “pathological stress” proceeding in a range of diseases—cancer, skeletal myogenesis, Alzheimer´s disease, rheumatoid arthritis, inflammatory bowel disease, autoimmune hepatitis and membranous glomerulonephritis. Morphine administration resulted in up-regulation of α-enolase in striatal neuronal cell cultures [47], but the change of this protein was not detected in hippocampus of morphine-treated rats by proteomic analysis of Bierczynska-Krzysik et al. [48].

Apart from protein changes *persisting* for 20 days after morphine withdrawal, decreased levels of tubulin α-1A chain, cofilin-1 and superoxide dismutase were found in PNS prepared from (±M10/−M20) rats (Table 3). Data of Marie-Claire et al. [49] indicated decreased mRNA and protein level of α-tubulin in rat striatum after chronic morphine treatment, whilst proteomic analysis of Bodzon-Kulakowska et al. [47] indicated the increase of α- and β-chains of tubulin in striatal neuronal cell cultures after morphine exposure. The full understanding of the functional meaning of down- or up-regulation of cytoskeleton protein tubulin α-1A will therefore require further attention.

The large decrease of superoxide dismutase (↓3.1-fold) in hippocampal samples (±M10/−M20) (Table 3) may be interpreted as the evidence of attenuated efficiency of hippocampus recovering from prolonged morphine effect in protection against oxidative stress. Superoxide dismutase, the enzyme catalyzing the conversion of superoxide radical O_2_^─^ to O_2_ and H_2_O_2_, represents an important antioxidant system protecting mammalian cells against oxidative damage [50,51]. This protection is necessary as morphine treatment was reported to modulate both enzymatic and non-enzymatic antioxidant defense systems and induce oxidative stress and apoptosis under *in vivo* conditions [52,53].

### Label-free quantification of rat hippocampus from animals exposed to morphine for 10 days (±M10); comparison with animals exposed to morphine and subsequently nurtured for 20 days in the absence of this drug (±M10/−M20)

With the aim to obtain a larger coverage of proteome changes elicited by morphine, we have also used the LFQ technique. The results supported those obtained by gel-based proteomic analysis. The LFQ identified 19 proteins with significantly changed expression level (≥1.7-fold) in (±M10) samples and this number was *increased* to 20 in (±M10/−M20) samples of hippocampus, Tables 5 and 6, Fig 9. Thus, *the lack of reversibility of chronic morphine* effect after 20 days of drug withdrawal was confirmed.

What type of underlying mechanism could cause this lack of reversibility? Opioids were reported to cause significant changes of glutamatergic transmission [54], neurogenesis [55], dendritic stability [56] and long-term potentiation [57–59]. Data of Cai et al. [60] indicated that morphine-treatment of mice for 6 days resulted in a decrease of excitatory synapse densities in parallel with an enhancement of densities of inhibitory synapses in hippocampus. This effect was mediated by μ-opioid receptors. In primary hippocampal neurons, the short-term exposure to morphine (0.5–24 h) induced an increase of intracellular levels of reactive oxygen species (ROS) generated by NADPH-oxidase. The increase of ROS was followed by an increase of endoplasmic reticulum stress markers and autophagy. Mattei et al. [12] suggested that i) hippocampus is a vulnerable part of brain susceptible to oxidative damage during aging and chronic stress, ii) prolonged withdrawal may lead to homeostatic changes heading for the restoration of the physiological norm and iii) abuse of morphine may lead to ROS-induced neurodegeneration and apoptosis.

The LFQ analysis of our withdrawal samples (Table 6) also confirmed the results of 2D-DIGE and 2D immunoblot analyses indicating the decrease of α-synuclein level (↓1.7-fold). The α-synuclein was described to participate in negative regulation of apoptosis, aging and oxidative stress [61].

In humans, heroin abuse was shown to increase oxidative stress [62]—plasma concentrations of lipid peroxides (LPO) and nitric oxide were increased in parallel with a decrease of antioxidants, vitamin C, vitamin E and beta-carotene. In erythrocytes, LPO were increased but the activity of antioxidant enzymes superoxide dismutase, catalase and glutathione peroxidase was decreased; in mice [63], heroin treatment resulted in a decrease of total antioxidant capacity in serum and activity of antioxidant enzymes superoxide dismutase, catalase and glutathione peroxidase in brain. Disturbance of pro-antioxidant balance was associated with oxidative damage of brain DNA, proteins and lipids. Addition of exogenous antioxidants attenuated the oxidative stress.

Results presented in our work extend these data as the down-regulation of superoxide dismutase protein level (↓3.1-fold) was detected in hippocampal samples collected from (±M10/−M20) rats (Table 3). As discussed above, the large decrease in the expression level of this anti-oxidant enzyme may be interpreted as a piece of supportive evidence for attenuated efficiency of rat hippocampus recovering from addiction to morphine. Protection against oxidative damage is decreased and return to physiological norm obscured.

## Conclusions

1. The 2D-DIGE analysis identified in the hippocampus of rats exposed to morphine for 10 days (+M10) six altered proteins when compared with samples prepared from control animals (−M10). In rats treated with morphine for 10 days and subsequently nurtured for 20 days in the absence of the drug (+M10/−M20), thirteen altered proteins were detected when compared with control animals (−M10/−M20). *Thus*, *the number of protein spots with changed expression level* (≥2-fold) *was* ***increased 2-fold after 20 days of abstinence***.
2. This result is just the opposite when compared with that detected in the forebrain cortex, where the number of altered proteins was decreased from 28 (±M10) to 14 (±M10/−M20) when determined in CBB-stained 2D gels or from 113 to 19 when determined by LFQ [10].
3. Noticeably, the altered expression level of four proteins identified in (±M10) samples (α-synuclein, β-synuclein, α-enolase and GAPDH) persisted for 20 days since the withdrawal of morphine.
4. Immunoblot analysis of 2D gels by specific antibodies oriented against α/β-synucleins and GAPDH confirmed data obtained by comparative 2D-DIGE and MALDI-TOF MS/MS.
5. When using LFQ analysis, nineteen proteins with significantly changed expression level (≥1.7-fold) were identified in group (+M10) when compared with group (−M10) and the number of altered proteins was increased to twenty in hippocampal samples collected from rats after 20 days of morphine withdrawal (±M10/−M20).
6. We conclude that the morphine-induced alteration of protein composition in rat hippocampus after cessation of drug supply proceeds in a ***different manner*** when compared with the forebrain cortex. In the forebrain cortex, the total number of altered proteins was **decreased** after 20 days without morphine, whilst in the hippocampus, it was **increased**. Thus, the two functionally distinct parts of CNS respond to the disturbance of the homeostatic balance caused by drug addiction in a different manner with the aim to restore the physiological norm.

## Supporting information

S1 Table MALDI-TOF MS/MS analysis of eight altered protein spots (with a complete list of peptides) in PNS prepared from hippocampus of rats exposed to morphine for 10 days and sacrificed 24 h after the last dose; *difference of protein composition in PNS samples prepared from groups (+M10) and (*−*M10)*. (DOCX)

S2 Table MALDI-TOF MS/MS analysis of fifteen altered protein spots (with a complete list of peptides) in PNS prepared from hippocampus of rats exposed to morphine for 10 days and sacrificed 20 days after the last dose; *difference of protein composition in PNS samples prepared from groups (+M10/−M20) and (*−*M10/−M20)*. (DOCX)

S1 File 1D-SDS-PAGE and immunoblotting of beta-actin. (DOCX)

S2 File 1D-SDS-PAGE and immunoblotting of GAPDH. (DOCX)

S3 File The ARRIVE guidelines checklist. (PDF)
