# Supplementary material for: Proteomic analysis of protein composition of rat hippocampus exposed to morphine for 10 days; comparison with animals after 20 days of morphine withdrawal
Source: PLoS One. 2020 Apr 15;15(4):e0231721. doi: 10.1371/journal.pone.0231721 (PMC7159219; doi:10.1371/journal.pone.0231721)
Supplement: S1 Table — (DOCX) [file pone.0231721.s001.docx]

**S1 Table: MALDI-TOF MS/MS** analysis of eight altered protein spots in PNS prepared from hippocampus of rats exposed to morphine for 10 days and sacrificed 24 h after the last dose; *difference of protein composition in PNS samples prepared from groups (+M10) and (*−*M10).*

| **Spot** | **Accession** | **Protein name** | **Mascot** | **Matched** | **Peptides** | **SC^a^** | **MW^b^** | **pI^c^** | **Change** |
| --- | --- | --- | --- | --- | --- | --- | --- | --- | --- |
|  | **number** |  | **score** | **peptides** |  | **[%]** | **(kDa)** |  | **(fold)** |
|  |  |  |  |  |  |  |  |  |  |
| **1** | SYUB_RAT | Beta-synuclein | 134 | 9 | K.EGVVAAAEK.T | 42 | 14.5 | 4.48 | ↓ 2.0 |
|  |  |  |  |  | K.EGVLYVGSK.T |  |  |  |  |
|  |  |  |  |  | K.EGVVQGVASVAEK.T |  |  |  |  |
|  |  |  |  |  | K.EGVVQGVASVAEK.T |  |  |  |  |
|  |  |  |  |  | K.EGVVQGVASVAEKTK.E |  |  |  |  |
|  |  |  |  |  | K.EQASHLGGAVFSGAGNIAAATGLVK.K |  |  |  |  |
|  |  |  |  |  | K.EQASHLGGAVFSGAGNIAAATGLVK.K |  |  |  |  |
|  |  |  |  |  | K.TKEQASHLGGAVFSGAGNIAAATGLVK.K |  |  |  |  |
|  |  |  |  |  | K.TKEQASHLGGAVFSGAGNIAAATGLVK.K |  |  |  |  |
| **2** | SYUA_RAT | Alpha-synuclein | 249 | 5 | K.EGVVHGVTTVAEK.T | 36 | 14.5 | 4.74 | ↓ 2.5 |
|  |  |  |  |  | K.TVEGAGNIAAATGFVK.K |  |  |  |  |
|  |  |  |  |  | K.TVEGAGNIAAATGFVK.K |  |  |  |  |
|  |  |  |  |  | K.TVEGAGNIAAATGFVK.K |  |  |  |  |
|  |  |  |  |  | K.TKEQVTNVGGAVVTGVTAVAQK.T |  |  |  |  |
| **3** | ACTB_RAT | Actin, cytoplasmic 1 (fragment) | 146 | 8 | R.HQGVMVGMGQK.D + 2 Deamidated (NQ); Oxidation (M) | 13 | 42.1 | 5.29 | ↑ 2.5 |
|  |  |  |  |  | R.AVFPSIVGRPR.H |  |  |  |  |
|  |  |  |  |  | R.AVFPSIVGRPR.H |  |  |  |  |
|  |  |  |  |  | R.AVFPSIVGRPR.H |  |  |  |  |
|  |  |  |  |  | K.IWHHTFYNELR.V |  |  |  |  |
|  |  |  |  |  | K.SYELPDGQVITIGNER.F |  |  |  |  |
|  |  |  |  |  | K.SYELPDGQVITIGNER.F |  |  |  |  |
|  |  |  |  |  | K.SYELPDGQVITIGNER.F |  |  |  |  |
| **4** | ENOA_RAT | Alpha-enolase | 117 | 6 | R.EIFDSR.G | 11 | 47.4 | 6.16 | ↑ 2.2 |
|  |  |  |  |  | R.EIFDSR.G |  |  |  |  |
|  |  |  |  |  | R.EIFDSR.G |  |  |  |  |
|  |  |  |  |  | K.YNQILR.I |  |  |  |  |
|  |  |  |  |  | R.AAVPSGASTGIYEALELR.D |  |  |  |  |
|  |  |  |  |  | K.AGYTDQVVIGMDVAASEFYR.A |  |  |  |  |
| **5** | ENOA_RAT | Alpha-enolase | 679 | 25 | K.GVPLYR.H | 44 | 47.4 | 6.16 | ↑ 2.2 |
|  |  |  |  |  | R.DNDKTR.F |  |  |  |  |
|  |  |  |  |  | R.EIFDSR.G |  |  |  |  |
|  |  |  |  |  | R.EIFDSR.G |  |  |  |  |
|  |  |  |  |  | K.YNQILR.I |  |  |  |  |
|  |  |  |  |  | K.YNQILR.I |  |  |  |  |
|  |  |  |  |  | R.IGAEVYHNLK.N |  |  |  |  |
|  |  |  |  |  | R.YITPDQLADLYK.S |  |  |  |  |
|  |  |  |  |  | K.LAQSNGWGVMVSHR.S |  |  |  |  |
|  |  |  |  |  | K.LAQSNGWGVMVSHR.S + Deamidated (NQ) |  |  |  |  |
|  |  |  |  |  | K.LAQSNGWGVMVSHR.S + Deamidated (NQ) |  |  |  |  |
|  |  |  |  |  | R.IGAEVYHNLKNVIK.E + 2 Deamidated (NQ) |  |  |  |  |
|  |  |  |  |  | K.VNQIGSVTESLQACK.L |  |  |  |  |
|  |  |  |  |  | R.AAVPSGASTGIYEALELR.D |  |  |  |  |
|  |  |  |  |  | R.AAVPSGASTGIYEALELR.D |  |  |  |  |
|  |  |  |  |  | K.LAMQEFMILPVGASSFR.E |  |  |  |  |
|  |  |  |  |  | K.LAMQEFMILPVGASSFR.E |  |  |  |  |
|  |  |  |  |  | K.LAMQEFMILPVGASSFR.E + Oxidation (M) |  |  |  |  |
|  |  |  |  |  | K.LAMQEFMILPVGASSFR.E + Oxidation (M) |  |  |  |  |
|  |  |  |  |  | K.DATNVGDEGGFAPNILENK.E |  |  |  |  |
|  |  |  |  |  | K.FTATAGIQVVGDDLTVTNPK.R |  |  |  |  |
|  |  |  |  |  | K.FTATAGIQVVGDDLTVTNPK.R |  |  |  |  |
|  |  |  |  |  | K.AGYTDQVVIGMDVAASEFYR.A |  |  |  |  |
|  |  |  |  |  | K.AGYTDQVVIGMDVAASEFYR.A |  |  |  |  |
|  |  |  |  |  | R.SGETEDTFIADLVVGLCTGQIK.T |  |  |  |  |
| **6** | DLDH_RAT | Dihydrolipoyl dehydrogenase, mitochondrial | 180 | 7 | K.FPFAANSR.A | 12 | 54.6 | 7.96 | ↑ 2.0 |
|  |  |  |  |  | K.ILGHKSTDR.I |  |  |  |  |
|  |  |  |  |  | R.VCHAHPTLSEAFR.E |  |  |  |  |
|  |  |  |  |  | K.AEVITCDVLLVCIGR.R |  |  |  |  |
|  |  |  |  |  | K.AEVITCDVLLVCIGR.R |  |  |  |  |
|  |  |  |  |  | K.AEVITCDVLLVCIGR.R |  |  |  |  |
|  |  |  |  |  | R.RPFTQNLGLEELGIELDPK.G |  |  |  |  |
| **7** | D3ZGY4_RAT | Glyceraldehyde-3-phosphate dehydrogenase | 230 | 7 | R.IGHLVTR.A | 15 | 36.1 | 7.63 | ↓ 2.3 |
|  |  |  |  |  | K.VGVNGFGR.I + Deamidated (NQ) |  |  |  |  |
|  |  |  |  |  | K.LTGMAFR.V + Oxidation (M) |  |  |  |  |
|  |  |  |  |  | R.VPTPNVSVVDLTCR.L |  |  |  |  |
|  |  |  |  |  | K.LISWYDNEYGYSNR.V |  |  |  |  |
|  |  |  |  |  | K.LISWYDNEYGYSNR.V |  |  |  |  |
|  |  |  |  |  | K.LISWYDNEYGYSNR.V |  |  |  |  |
| **8** | D3ZGY4_RAT | Glyceraldehyde-3-phosphate dehydrogenase | 306 | 13 | K.LTGMAFR.V | 18 | 36.1 | 7.63 | ↓ 2.3 |
|  |  |  |  |  | K.LTGMAFR.V |  |  |  |  |
|  |  |  |  |  | K.VGVNGFGR.I |  |  |  |  |
|  |  |  |  |  | K.VGVNGFGR.I + Deamidated (NQ) |  |  |  |  |
|  |  |  |  |  | K.VGVNGFGR.I + Deamidated (NQ) |  |  |  |  |
|  |  |  |  |  | R.VPTPNVSVVDLTCR.L |  |  |  |  |
|  |  |  |  |  | R.VPTPNVSVVDLTCR.L |  |  |  |  |
|  |  |  |  |  | K.LVINGKPITIFQER.D |  |  |  |  |
|  |  |  |  |  | K.LVINGKPITIFQER.D + Deamidated (NQ) |  |  |  |  |
|  |  |  |  |  | K.LVINGKPITIFQER.D + Deamidated (NQ) |  |  |  |  |
|  |  |  |  |  | K.LISWYDNEYGYSNR.V |  |  |  |  |
|  |  |  |  |  | K.LISWYDNEYGYSNR.V |  |  |  |  |
|  |  |  |  |  | K.LVINGKPITIFQERDPANIK.W + Deamidated (NQ) |  |  |  |  |
|  |  |  |  |  |  |  |  |  |  |

*^a^* sequence coverage, *^b^* theoretical molecular weight, *^c^* theoretical isoelectric point
