# Supplementary material for: Proteomic analysis of protein composition of rat hippocampus exposed to morphine for 10 days; comparison with animals after 20 days of morphine withdrawal
Source: PLoS One. 2020 Apr 15;15(4):e0231721. doi: 10.1371/journal.pone.0231721 (PMC7159219; doi:10.1371/journal.pone.0231721)
Supplement: S2 Table — (DOCX) [file pone.0231721.s002.docx]

**S2 Table: MALDI-TOF MS/MS** analysis of fifteen altered protein spots in PNS prepared from hippocampus of rats exposed to morphine for 10 days and sacrificed 20 days after the last dose; *difference of protein composition in PNS samples prepared from groups (+M10/−M20) and (*−*M10/−M20).*

| **Spot** | **Accession** | **Protein name** | **Mascot** | **Matched** | **Peptides** | **SC^a^** | **MW^b^** | **pI^c^** | **Change** |
| --- | --- | --- | --- | --- | --- | --- | --- | --- | --- |
|  | **number** |  | **score** | **peptides** |  | **[%]** | **(kDa)** |  | **(fold)** |
|  |  |  |  |  |  |  |  |  |  |
| **1** | SYUB_RAT | Beta-synuclein | 96 | 5 | K.EGVVQGVASVAEK.T | 29 | 14.5 | 4.48 | ↓ 2.4 |
|  |  |  |  |  | K.EQASHLGGAVFSGAGNIAAATGLVK.K |  |  |  |  |
|  |  |  |  |  | K.TKEQASHLGGAVFSGAGNIAAATGLVK.K |  |  |  |  |
|  |  |  |  |  | K.TKEQASHLGGAVFSGAGNIAAATGLVK.K |  |  |  |  |
|  |  |  |  |  | K.TKEQASHLGGAVFSGAGNIAAATGLVK.K |  |  |  |  |
| **2** | SYUA_RAT | Alpha-synuclein | 249 | 5 | K.EGVVHGVTTVAEK.T | 36 | 14.5 | 4.74 | ↓ 5.3 |
|  |  |  |  |  | K.TVEGAGNIAAATGFVK.K |  |  |  |  |
|  |  |  |  |  | K.TVEGAGNIAAATGFVK.K |  |  |  |  |
|  |  |  |  |  | K.TVEGAGNIAAATGFVK.K |  |  |  |  |
|  |  |  |  |  | K.TKEQVTNVGGAVVTGVTAVAQK.T |  |  |  |  |
| **3** | TBA1A_RAT | Tubulin alpha-1A chain | 298 | 19 | K.YMACCLLYR.G | 40 | 50.8 | 4.94 | ↓ 4.5 |
|  |  |  |  |  | R.TIQFVDWCPTGFK.V |  |  |  |  |
|  |  |  |  |  | R.AVFVDLEPTVIDEVR.T |  |  |  |  |
|  |  |  |  |  | R.AVFVDLEPTVIDEVR.T |  |  |  |  |
|  |  |  |  |  | R.NLDIERPTYTNLNR.L |  |  |  |  |
|  |  |  |  |  | R.IHFPLATYAPVISAEK.A |  |  |  |  |
|  |  |  |  |  | R.IHFPLATYAPVISAEK.A |  |  |  |  |
|  |  |  |  |  | K.VGINYQPPTVVPGGDLAK.V |  |  |  |  |
|  |  |  |  |  | K.VGINYQPPTVVPGGDLAK.V |  |  |  |  |
|  |  |  |  |  | R.GHYTIGKEIIDLVLDR.I |  |  |  |  |
|  |  |  |  |  | R.GHYTIGKEIIDLVLDR.I |  |  |  |  |
|  |  |  |  |  | R.AVCMLSNTTAIAEAWAR.L |  |  |  |  |
|  |  |  |  |  | R.AVCMLSNTTAIAEAWAR.L + Oxidation (M) |  |  |  |  |
|  |  |  |  |  | K.TIGGGDDSFNTFFSETGAGK.H |  |  |  |  |
|  |  |  |  |  | R.GHYTIGKEIIDLVLDRIR.K |  |  |  |  |
|  |  |  |  |  | R.GHYTIGKEIIDLVLDRIR.K |  |  |  |  |
|  |  |  |  |  | R.FDGALNVDLTEFQTNLVPYPR.I |  |  |  |  |
|  |  |  |  |  | R.QLFHPEQLITGKEDAANNYAR.G |  |  |  |  |
|  |  |  |  |  | R.QLFHPEQLITGKEDAANNYAR.G |  |  |  |  |
| **5** | PDIA3_RAT | Protein disulfide-isomerase A3 | 117 | 10 | R.TEDEFKK.F | 13 | 57.0 | 5.88 | ↓ 2.2 |
|  |  |  |  |  | R.TEDEFKK.F |  |  |  |  |
|  |  |  |  |  | K.AASNLRDNYR.F + 2 Deamidated (NQ) |  |  |  |  |
|  |  |  |  |  | K.AASNLRDNYR.F + 2 Deamidated (NQ) |  |  |  |  |
|  |  |  |  |  | R.ELNDFISYLQR.E |  |  |  |  |
|  |  |  |  |  | K.NTKGSNYWRNR.V + 2 Deamidated (NQ) |  |  |  |  |
|  |  |  |  |  | R.FLQEYFDGNLKR.Y |  |  |  |  |
|  |  |  |  |  | R.FLQEYFDGNLKR.Y |  |  |  |  |
|  |  |  |  |  | K.MDATANDVPSPYEVK.G + Deamidated (NQ); Oxidation (M) |  |  |  |  |
|  |  |  |  |  | K.MDATANDVPSPYEVK.G + Deamidated (NQ); Oxidation (M) |  |  |  |  |
| **6** | ATP5H_RAT | ATP synthase subunit d, mitochondrial | 305 | 8 | K.SWNETFHTR.L | 31 | 18.8 | 6.17 | ↓ 2.0 |
|  |  |  |  |  | K.SWNETFHTR.L |  |  |  |  |
|  |  |  |  |  | K.NCAQFVTGSQAR.V |  |  |  |  |
|  |  |  |  |  | K.NCAQFVTGSQAR.V |  |  |  |  |
|  |  |  |  |  | R.KYPYWPHQPIENL.- |  |  |  |  |
|  |  |  |  |  | R.KYPYWPHQPIENL.- |  |  |  |  |
|  |  |  |  |  | R.LASLSEKPPAIDWAYYR.A |  |  |  |  |
|  |  |  |  |  | R.LASLSEKPPAIDWAYYR.A |  |  |  |  |
| **7** | SODC_RAT | Superoxide dismutase [Cu-Zn] | 81 | 6 | K.KHGGPADEER.H | 31 | 16.1 | 5.88 | ↓ 3.1 |
|  |  |  |  |  | K.DGVANVSIEDR.V |  |  |  |  |
|  |  |  |  |  | K.DGVANVSIEDR.V |  |  |  |  |
|  |  |  |  |  | R.VISLSGEHSIIGR.T |  |  |  |  |
|  |  |  |  |  | K.GDGPVQGVIHFEQK.A |  |  |  |  |
|  |  |  |  |  | K.GDGPVQGVIHFEQK.A |  |  |  |  |
| **8** | DPYL2_RAT | Dihydropyrimidinase-related protein 2 | 237 | 9 | R.IVAPPGGR.A | 17 | 62.6 | 5.95 | ↓ 2.4 |
|  |  |  |  |  | K.VFNLYPR.K |  |  |  |  |
|  |  |  |  |  | K.VFNLYPR.K |  |  |  |  |
|  |  |  |  |  | K.VFNLYPR.K |  |  |  |  |
|  |  |  |  |  | R.KPFPDFVYK.R |  |  |  |  |
|  |  |  |  |  | K.IVLEDGTLHVTEGSGR.Y |  |  |  |  |
|  |  |  |  |  | R.FQLTDSQIYEVLSVIR.D |  |  |  |  |
|  |  |  |  |  | R.NLHQSGFSLSGAQIDDNIPR.R |  |  |  |  |
|  |  |  |  |  | R.DIGAIAQVHAENGDIIAEEQQR.I |  |  |  |  |
| **9** | ENOA_RAT | Alpha-enolase | 694 | 20 | K.GVPLYR.H | 28 | 47.4 | 6.16 | ↑ 3.1 |
|  |  |  |  |  | R.EIFDSR.G |  |  |  |  |
|  |  |  |  |  | R.EIFDSR.G |  |  |  |  |
|  |  |  |  |  | K.YNQILR.I |  |  |  |  |
|  |  |  |  |  | K.YNQILR.I |  |  |  |  |
|  |  |  |  |  | R.IGAEVYHNLK.N |  |  |  |  |
|  |  |  |  |  | R.YITPDQLADLYK.S |  |  |  |  |
|  |  |  |  |  | K.LAQSNGWGVMVSHR.S |  |  |  |  |
|  |  |  |  |  | K.LAQSNGWGVMVSHR.S + Deamidated (NQ) |  |  |  |  |
|  |  |  |  |  | K.LAQSNGWGVMVSHR.S + Deamidated (NQ) |  |  |  |  |
|  |  |  |  |  | K.VNQIGSVTESLQACK.L |  |  |  |  |
|  |  |  |  |  | K.VNQIGSVTESLQACK.L |  |  |  |  |
|  |  |  |  |  | R.AAVPSGASTGIYEALELR.D |  |  |  |  |
|  |  |  |  |  | R.AAVPSGASTGIYEALELR.D |  |  |  |  |
|  |  |  |  |  | K.LAMQEFMILPVGASSFR.E |  |  |  |  |
|  |  |  |  |  | K.LAMQEFMILPVGASSFR.E |  |  |  |  |
|  |  |  |  |  | K.LAMQEFMILPVGASSFR.E + Oxidation (M) |  |  |  |  |
|  |  |  |  |  | K.LAMQEFMILPVGASSFR.E + Oxidation (M) |  |  |  |  |
|  |  |  |  |  | K.AGYTDQVVIGMDVAASEFYR.A |  |  |  |  |
|  |  |  |  |  | K.AGYTDQVVIGMDVAASEFYR.A |  |  |  |  |
| **10** | EFTU_RAT | Elongation factor Tu, mitochondrial | 242 | 8 | K.YEEIDNAPEER.A | 13 | 49.9 | 7.23 | ↓ 2.2 |
|  |  |  |  |  | K.YEEIDNAPEER.A |  |  |  |  |
|  |  |  |  |  | K.LLDAVDTYIPVPTR.D |  |  |  |  |
|  |  |  |  |  | K.LLDAVDTYIPVPTR.D |  |  |  |  |
|  |  |  |  |  | R.GITINAAHVEYSTAAR.H |  |  |  |  |
|  |  |  |  |  | R.GITINAAHVEYSTAAR.H |  |  |  |  |
|  |  |  |  |  | R.DLEKPFLLPVESVYSIPGR.G |  |  |  |  |
|  |  |  |  |  | R.DLEKPFLLPVESVYSIPGR.G |  |  |  |  |
| **11** | D3ZGY4_RAT | Glyceraldehyde-3-phosphate dehydrogenase | 109 | 9 | K.VGVNGFGR.I | 9 | 36.1 | 7.63 | ↓ 2.4 |
|  |  |  |  |  | K.VGVNGFGR.I + Deamidated (NQ) |  |  |  |  |
|  |  |  |  |  | K.VGVNGFGR.I + Deamidated (NQ) |  |  |  |  |
|  |  |  |  |  | K.QVAEGPLK.G + Deamidated (NQ) |  |  |  |  |
|  |  |  |  |  | M.VKVGVNGFGR.I |  |  |  |  |
|  |  |  |  |  | M.VKVGVNGFGR.I |  |  |  |  |
|  |  |  |  |  | M.VKVGVNGFGR.I + Deamidated (NQ) |  |  |  |  |
|  |  |  |  |  | K.LISWYDNEYGYSNR.V |  |  |  |  |
|  |  |  |  |  | K.LISWYDNEYGYSNR.V |  |  |  |  |
| **12** | D3ZGY4_RAT | Glyceraldehyde-3-phosphate dehydrogenase | 273 | 6 | K.VGVNGFGR.I + Deamidated (NQ) | 10 | 36.1 | 7.63 | ↓ 2.3 |
|  |  |  |  |  | K.VGVNGFGR.I + Deamidated (NQ) |  |  |  |  |
|  |  |  |  |  | R.VPTPNVSVVDLTCR.L |  |  |  |  |
|  |  |  |  |  | R.VPTPNVSVVDLTCR.L |  |  |  |  |
|  |  |  |  |  | K.LISWYDNEYGYSNR.V |  |  |  |  |
|  |  |  |  |  | K.LISWYDNEYGYSNR.V |  |  |  |  |
| **13** | D3ZGY4_RAT | Glyceraldehyde-3-phosphate dehydrogenase | 316 | 13 | K.VGVNGFGR.I | 20 | 36.1 | 7.63 | ↓ 4.1 |
|  |  |  |  |  | K.VGVNGFGR.I + Deamidated (NQ) |  |  |  |  |
|  |  |  |  |  | K.VGVNGFGR.I + Deamidated (NQ) |  |  |  |  |
|  |  |  |  |  | K.TVDGPSGKLWR.D |  |  |  |  |
|  |  |  |  |  | K.TVDGPSGKLWR.D |  |  |  |  |
|  |  |  |  |  | R.VPTPNVSVVDLTCR.L |  |  |  |  |
|  |  |  |  |  | R.VPTPNVSVVDLTCR.L |  |  |  |  |
|  |  |  |  |  | K.LVINGKPITIFQER.D |  |  |  |  |
|  |  |  |  |  | K.LVINGKPITIFQER.D |  |  |  |  |
|  |  |  |  |  | K.LVINGKPITIFQER.D + Deamidated (NQ) |  |  |  |  |
|  |  |  |  |  | K.LISWYDNEYGYSNR.V |  |  |  |  |
|  |  |  |  |  | K.LISWYDNEYGYSNR.V |  |  |  |  |
|  |  |  |  |  | K.LVINGKPITIFQERDPANIK.W + Deamidated (NQ) |  |  |  |  |
| **14** | COF1_RAT | Cofilin-1 | 346 | 9 | K.AVLFCLSEDKK.N | 40 | 18.7 | 8.22 | ↓ 2.8 |
|  |  |  |  |  | K.AVLFCLSEDKK.N |  |  |  |  |
|  |  |  |  |  | R.YALYDATYETK.E |  |  |  |  |
|  |  |  |  |  | R.YALYDATYETK.E |  |  |  |  |
|  |  |  |  |  | K.LGGSAVISLEGKPL.- |  |  |  |  |
|  |  |  |  |  | K.HELQANCYEEVKDR.C |  |  |  |  |
|  |  |  |  |  | K.HELQANCYEEVKDR.C |  |  |  |  |
|  |  |  |  |  | K.KEDLVFIFWAPESAPLK.S |  |  |  |  |
|  |  |  |  |  | K.KEDLVFIFWAPESAPLK.S |  |  |  |  |
| **15** | ALDOA_RAT | Fructose-bisphosphate aldolase A | 255 | 24 | K.ELADIAHR.I | 53 | 39.8 | 8.31 | ↓ 2.8 |
|  |  |  |  |  | K.DGADFAKWR.C |  |  |  |  |
|  |  |  |  |  | K.AAQEEYIKR.A |  |  |  |  |
|  |  |  |  |  | K.AAQEEYIKR.A |  |  |  |  |
|  |  |  |  |  | K.ADDGRPFPQVIK.S |  |  |  |  |
|  |  |  |  |  | K.ADDGRPFPQVIK.S |  |  |  |  |
|  |  |  |  |  | R.ALQASALKAWGGKK.E |  |  |  |  |
|  |  |  |  |  | R.ALQASALKAWGGKK.E |  |  |  |  |
|  |  |  |  |  | M.PHPYPALTPEQKK.E |  |  |  |  |
|  |  |  |  |  | R.LQSIGTENTEENRR.F |  |  |  |  |
|  |  |  |  |  | K.RLQSIGTENTEENRR.F |  |  |  |  |
|  |  |  |  |  | K.RLQSIGTENTEENRR.F |  |  |  |  |
|  |  |  |  |  | K.FSNEEIAMATVTALRR.T |  |  |  |  |
|  |  |  |  |  | K.FSNEEIAMATVTALRR.T + Oxidation (M) |  |  |  |  |
|  |  |  |  |  | R.VNPCIGGVILFHETLYQK.A + 2 Deamidated (NQ) |  |  |  |  |
|  |  |  |  |  | K.YTPSGQSGAAASESLFISNHAY.- |  |  |  |  |
|  |  |  |  |  | K.YTPSGQSGAAASESLFISNHAY.- |  |  |  |  |
|  |  |  |  |  | K.GVVPLAGTNGETTTQGLDGLSER.C |  |  |  |  |
|  |  |  |  |  | K.GVVPLAGTNGETTTQGLDGLSER.C |  |  |  |  |
|  |  |  |  |  | M.PHPYPALTPEQKKELADIAHR.I |  |  |  |  |
|  |  |  |  |  | R.QLLLTADDRVNPCIGGVILFHETLYQK.A |  |  |  |  |
|  |  |  |  |  | R.QLLLTADDRVNPCIGGVILFHETLYQK.A |  |  |  |  |
|  |  |  |  |  | R.YASICQQNGIVPIVEPEILPDGDHDLKR.C |  |  |  |  |
|  |  |  |  |  | R.YASICQQNGIVPIVEPEILPDGDHDLKR.C |  |  |  |  |
|  |  |  |  |  |  |  |  |  |  |

*^a^* sequence coverage, *^b^* theoretical molecular weight, *^c^* theoretical isoelectric point
