# Supplementary material for: Proteomic analysis of protein composition of rat hippocampus exposed to morphine for 10 days; comparison with animals after 20 days of morphine withdrawal
Source: PLoS One. 2020 Apr 15;15(4):e0231721. doi: 10.1371/journal.pone.0231721 (PMC7159219; doi:10.1371/journal.pone.0231721)
Supplement: S1 File — (DOCX) [file pone.0231721.s003.docx]

**1D-SDS-PAGE and immunoblotting of beta-actin**

The aliquots of PNS samples (−M10, +M10 and −M10/−M20, +M10/−M20) were mixed 1:1 with 2-fold concentrated Laemmli buffer (SLB) with 1 mM dithiothreitol (DTT) and heated for 3 min at 100°C. Standard (10% w/v acrylamide/0.26% w/v bis-acrylamide) SDS-PAGE electrophoresis was run at 200 V for 45 min. Molecular mass determinations were based on pre-stained molecular mass markers (Sigma, SDS 7B).

After SDS-PAGE, proteins were transferred to nitrocellulose (Protran BA 83, GE Healthcare) and blocked for 1 h at room temperature in 5% (w/v) low-fat milk in TBS-Tween buffer (10 mM Tris-HCl, pH 8.0, 150 mM NaCl, 0.1% (v/v) Tween 20). Primary antibody against beta-actin (bs-0061R, 1:5000 dilution) was added in TBS-Tween containing 1% (w/v) low-fat milk and incubated for at least 2 h, then removed and the membrane was washed extensively (3 × 10 min) in TBS-Tween. Secondary antibody (donkey anti-rabbit IgG-HRP NA934V, GE Healthcare, 1:10000 dilution) was diluted in TBS-Tween containing 1% (w/v) low-fat milk, applied for 1 h and after three 10 min washes, the blots were developed by ECL technique using Super Signal West Dura (Pierce) as substrate. The developed blots were scanned with an imaging densitometer Epson Perfection 4990 Photo and quantified by Aida Image Analyzer v. 3.28 (Ray test).


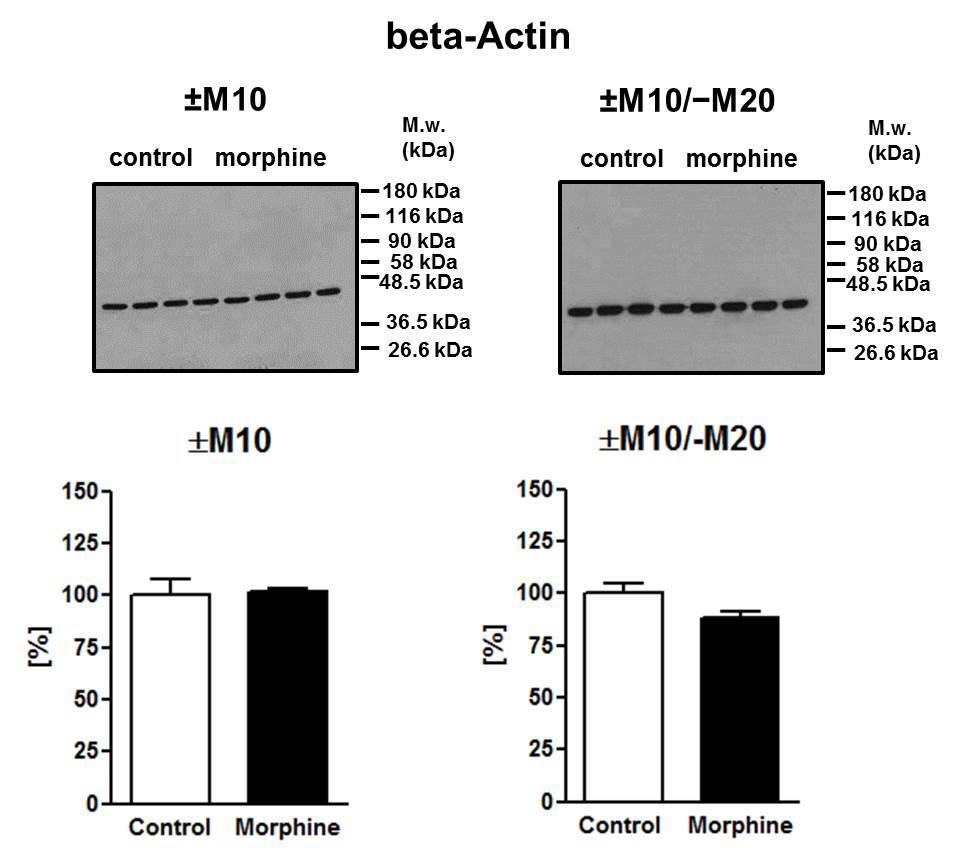


**1D immunoblot analysis of beta-actin in PNS prepared from rat hippocampus of experimental groups (±M10) and (±M10/─M20).** PNS proteins (20 μg per lane) were resolved under dissociated conditions (+DTT) by standard SDS-PAGE in 10% w/v acrylamide/0.26% w/v bis-acrylamide gels, and immunoblotted. The immunoblot signal of beta-actin was detected by bs-0061R (beta-actin polyclonal antibody) purchased from Bioss Antibodies. Analysis of beta-actin was based on signals collected from three immunoblots. The significance of the difference between the groups of samples (−M10, +M10 and −M10/−M20, +M10/−M20) was analyzed by Student´s *t*-test using GraphPad*Prizm4* and was not significant (NS, p>0.05).
