# Supplementary material for: Proteomic analysis of protein composition of rat hippocampus exposed to morphine for 10 days; comparison with animals after 20 days of morphine withdrawal
Source: PLoS One. 2020 Apr 15;15(4):e0231721. doi: 10.1371/journal.pone.0231721 (PMC7159219; doi:10.1371/journal.pone.0231721)
Supplement: S2 File — (DOCX) [file pone.0231721.s004.docx]

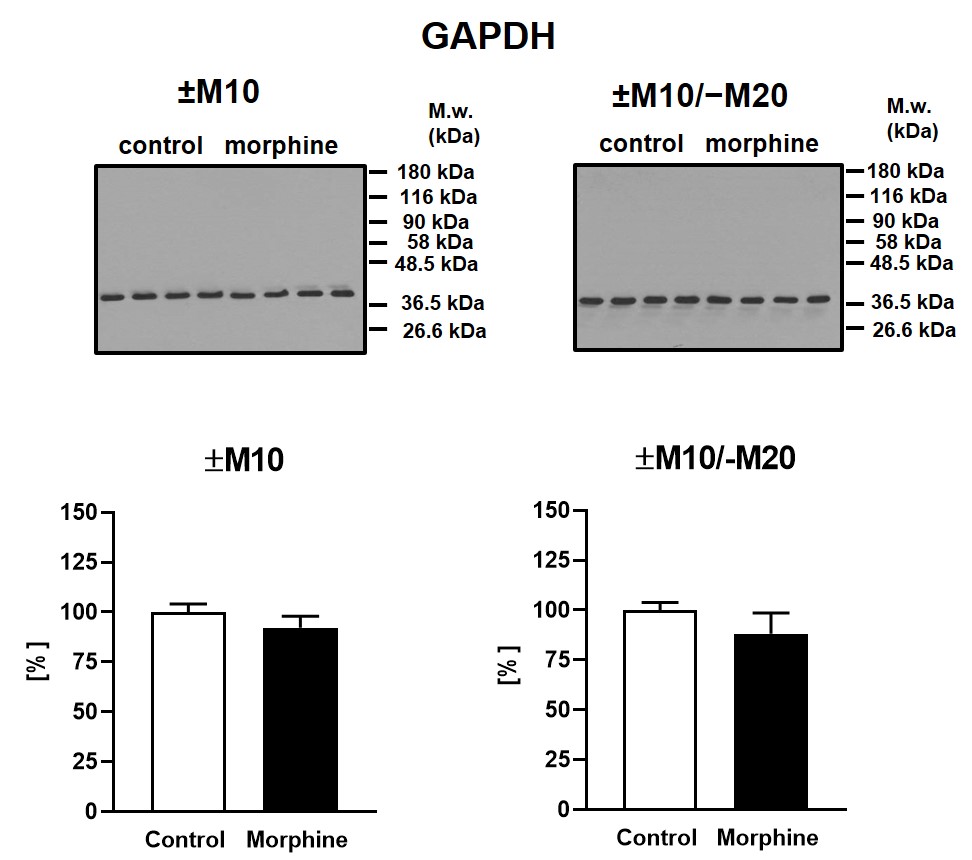


**1D immunoblot analysis of GAPDH in PNS prepared from rat hippocampus of experimental groups (±M10) and (±M10/─M20).** PNS proteins (10 μg per lane) were resolved under dissociated conditions (+DTT) by standard SDS-PAGE in 10% w/v acrylamide/0.26% w/v bis-acrylamide gels, and immunoblotted. The immunoblot signal of GAPDH was detected by sc-25778 (1:5000 dilution) purchased from Santa Cruz. Analysis of GAPDH was based on signals collected from three immunoblots. The significance of the difference between the groups of samples (−M10, +M10 and −M10/−M20, +M10/−M20) was analyzed by Student´s *t*-test using GraphPad*Prism4* and was not significant (NS, p>0.05).
